# Identification of hub genes and their novel diagnostic and prognostic significance in pancreatic adenocarcinoma

**DOI:** 10.20892/j.issn.2095-3941.2020.0516

**Published:** 2021-08-17

**Authors:** Duo Zuo, Yongzi Chen, Xinwei Zhang, Zhuozhi Wang, Wenna Jiang, Fan Tang, Runfen Cheng, Yi Sun, Lu Sun, Li Ren, Rui Liu

**Affiliations:** 1 Department of Clinical Laboratory, Tianjin Medical University Cancer Institute and Hospital, National Clinical Research Center for Cancer, Key Laboratory of Cancer Prevention and Therapy, Tianjin, Tianjin’s Clinical Research Center for Cancer, Tianjin 300060, China; 2 Department of Tumor Cell Biology, Tianjin Medical University Cancer Institute and Hospital, National Clinical Research Center for Cancer, Key Laboratory of Cancer Prevention and Therapy, Tianjin, Tianjin’s Clinical Research Center for Cancer, Tianjin 300060, China; 3 School of Biomedical Engineering, Tianjin Medical University, Tianjin 300070, China; 4 Department of Pathology, Tianjin Huanhu Hospital, Tianjin 300350, China; 5 Department of Pathology, Tianjin Medical University Cancer Institute and Hospital, National Clinical Research Center for Cancer, Key Laboratory of Cancer Prevention and Therapy, Tianjin, Tianjin’s Clinical Research Center for Cancer, Tianjin 300060, China; 6 School of Pharmacy, Tianjin Medical University, Tianjin 300070, China; 7 Department of Gastrointestinal Medical Oncology, Tianjin Medical University Cancer Institute and Hospital, National Clinical Research Center for Cancer, Key Laboratory of Cancer Prevention and Therapy, Tianjin, Tianjin’s Clinical Research Center for Cancer, Tianjin 300060, China

**Keywords:** *RSAD2*, *SMC4*, *DLGAP5*, *ISG15*, pancreatic adenocarcinoma

## Abstract

**Objective:**

The main reasons for the poor prognoses of pancreatic adenocarcinoma (PA) patients are rapid early-stage progression, advanced stage metastasis, and chemotherapy resistance. Identification of novel diagnostic and prognostic biomarkers of PA is therefore urgently needed.

**Methods:**

Three mRNA microarray datasets were obtained from the Gene Expression Omnibus database to select differentially expressed genes (DEGs). Gene Ontology (GO) and Kyoto Encyclopedia of Genes and Genomes pathway enrichment analyses for hub genes were performed using DAVID. Correlations between expression levels of hub genes and cancer-infiltrating immune cells were investigated by TIMER. Cox proportional hazard regression analyses were also performed. Serum hub genes were screened using the HPA platform and verified for diagnostic value using ELISAs.

**Results:**

We identified 59 hub genes among 752 DEGs. GO analysis indicated that these 59 hub genes were mainly involved in the defense response to viruses and the type I interferon signaling pathway. We also discovered that *RSAD2* and *SMC4* were associated with immune cell infiltration in the PA microenvironment. Additionally, *DLGAP5* mRNA might be used as an independent risk factor for the prognoses of PA patients. Furthermore, the protein encoded by *ISG15*, which exists in peripheral blood, was validated as a potential diagnostic biomarker that distinguished PA patients from healthy controls (area under the curve: 0.902, 95% confidence interval: 0.819–0.961).

**Conclusions:**

Our study suggested that *RSAD2* and *SMC4* were associated with immune cell infiltration in the PA microenvironment, while *DLGAP5* mRNA expression might be an independent risk factor for the survival prognoses of PA patients. Moreover, ELISAs indicated that serum ISG15 could be a potential novel diagnostic biomarker for PA.

## Introduction

Pancreatic adenocarcinoma (PA) is a highly lethal tumor with a poor prognosis. Despite recent advances in diagnosis and treatment, its 5-year survival rate remains at 8%^1^. PA is predicted to be the second leading cause of cancer-related mortality by 2030^2^. Many PA patients postoperatively develop recurrences typically within 2 years and die within 5 years of recurrence^3–5^. Therefore, new requirements for diagnosis and treatment strategies have been proposed to improve the survival time of PA patients.

Recently, the increased use of immunotherapies has helped in the treatment of advanced PA patients. However, with feedback from clinical trials, PA is considered to be a type of “immune cold” tumor^6^. The outcome of many PA patients has not been significantly improved by immunotherapy, which poses a new challenge for immunotherapy strategies^7^. Oncolytic viruses (OVs) are emerging immunotherapeutics for many cancers. Tumor-targeted OVs are usually engineered viruses that can selectively infect, replicate in, and lyse tumor cells, and also induce immune responses specifically against tumor cells^8^. OVs can also change the immunogenicity of the tumor microenvironment from an immunosuppressed (immunologically “cold”) state to an immunoactivated (immunologically “hot”) state^9^. The oncolytic herpesvirus, talimogene laherparepvec, was approved by the United States Food and Drug Administration and European Medicines Agency for advanced melanoma treatment in 2015. In addition, several OVs have been used in clinical trials of PA^10,11^. However, studies have found that some PA patients develop drug resistance due to the type I interferon (IFN) responses of the host that restrict viral replication^12^. The products of IFN-stimulated genes (ISGs) have been reported to contribute to the resistance of PA cells to OVs^13–15^. There is therefore an urgent need to identify specific defects in genes involved in IFN signaling pathways as potential biomarkers to classify candidate patients for effective oncolytic therapy.

The molecular study of carcinogenesis and progression is very important for patient therapy, diagnosis, and prognoses^16,17^. Using studies of the carcinogenesis and progression of PA, increasing attention has been directed toward abnormal expressions and mutations of genes^18–21^. In recent years, many studies have screened cancer-related differentially expressed genes (DEGs) by microarray technology. Furthermore, a microarray technique has been used to detect PA cells infected with an OV, and it was found that the expression levels of certain genes were significantly altered before and after viral infection, so these genes could be used as potential biomarkers to predict the effectiveness of oncolytic therapy^22^. However, the clinical sample size for PA is smaller than that of other common cancers^23^, leading to controversial results between different microarray chip analyses^24^. Thus, the integration of microarray technology and bioinformatics analysis technology is useful in identifying novel DEGs and functional pathways related to the occurrence and development of PA, as well as clinical responses to therapies^25–27^.

In the current study, we identified 752 DEGs between PA tissues and noncancerous tissues extracted from the Gene Expression Omnibus (GEO) database, among which 59 DEGs were identified as hub genes using Protein-Protein Interaction (PPI) network analysis. Gene Ontology (GO) and Kyoto Encyclopedia of Genes and Genomes (KEGG) pathway enrichment analyses were used to characterize the hub genes at the molecular level. These hub genes were mainly enriched in the defense response to virus and the type I IFN signaling pathways. Furthermore, the correlation between hub genes and immune cell infiltration in the microenvironment was investigated by the Tumor Immune Estimation Resource (TIMER) webserver to provide new insight for OV therapy. More importantly, we screened potential biomarkers related to the diagnosis and prognosis of PA from 59 hub genes.

## Materials and methods

### Microarray data

The mRNA microarray datasets, GSE15471, GSE16515, and GSE71989, based on the GPL570 platform, (HG-U133_Plus_2 Affymetrix Human Genome U133 Plus 2.0 Array) (Affymetrix, Santa Clara, CA, USA) were obtained from the GEO database (http://www.ncbi.nlm.nih.gov/geo), which is a public functional genomics data repository containing microarray- and chip-based data of gene profiles^28^. In summary, GSE15471^29^ contained 36 PA tissue samples and 36 normal pancreatic tissue samples. GSE16515^30^ contained 36 PA tissue samples and 16 normal pancreatic tissue samples. Thirteen PA tissue samples and 8 normal pancreatic tissue samples were included in the GSE71989^31^ dataset.

### Identification of DEGs

The online GEO2R tool (http://www.ncbi.nlm.nih.gov/geo/geo2r/) was used to screen DEGs between PA and normal pancreatic tissues. During analysis using GEO2R, the Benjamini & Hochberg (false discovery rate) method was used to adjust the *P*-values to balance the results of statistically significant genes and the limitations of false positives. Furthermore, a |log fold change (FC)| > 1 and adjusted *P*-value < 0.01 were further set as the cut-off criteria for screening DEGs.

### GO functional and KEGG pathway enrichment analyses of DEGs

GO functional analysis is a useful bioinformatics tool for annotating genes and analyzing characteristic biological attributes for high-throughput genome or transcriptome data^32^. GO terms contain 3 main categories: biological process (BP), cellular component (CC), and molecular function (MF). KEGG (http://www.kegg.jp), an online database resource, including defined and associated gene sets and their pathways, was used for identifying high level functions and biological systems^33^. The GO functional analyses and KEGG pathway enrichment analyses of DEGs were generated by using Database for Annotation, Visualization and Integrated Discovery (DAVID, version 6.8, http://david.ncifcrf.gov/)^34^. *P* < 0.05 and false discovery rate ≤ 0.01 were set as the filter criteria.

### Visualized PPI network construction and hub gene selection

Search Tool for the Retrieval of Interacting Genes (STRING, version 11.0, http://string-db.org)^35^ was used to predict a PPI network and evaluate the comprehensive information of proteins. In this study, a PPI network of all candidate DEGs was generated using the STRING website, and each interaction between proteins with a reliability threshold of a combined score of > 0.4 was considered statistically significant. The PPI network was further visualized by Cytoscape (version 3.7.1, http://www.cytoscape.org/)^36^. The Molecular Complex Detection (MCODE, version 1.5.1) plugin of Cytoscape was used to obtain the most densely connected module (the most significant clustering module) in the visible PPI network. The filter criteria for the most densely connected module were set as follows: “MCODE scores > 5”, “node score cut-off = 0.2”, “k-score = 2”, “degree cut-off = 2”, and “Max depth = 100”. The hub genes were selected with degrees ≥ 20 in the most densely connected module.

### TIMER database analysis

TIMER is a database that provides a deconvolution algorithm for systematic analysis to investigate the correlation between gene expressions and infiltrating immune cells, including B cells, CD4+ T cells, CD8+ T cells, macrophages, neutrophils, and dendritic cells^37^. Correlations between the expression levels of hub genes and the infiltrating immune cells were analyzed using correlation modules. Moreover, the association between hub genes and tumor purity was also determined in the microenvironment of PA. The scatterplots were displayed by the TIMER web tool for correlation visualization. *P* < 0.05 was considered to indicate a statistically significant correlation.

### Prognostic analyses of hub genes

The correlations of hub gene expression with overall survival (OS) and disease-free survival (DFS) were displayed by Kaplan-Meier curves from The Cancer Genome Atlas (TCGA) database in the Gene Expression Profiling Interactive Analysis (GEPIA, http://gepia.cancer-pku.cn/) platform^38^. The median expression of the hub gene was set as the cut-off for demarcating high and low expression groups. A log-rank *P*-value < 0.05 was considered statistically significant. In addition, using the cBio Cancer Genomics Portal (cBioPortal, http://cbioportal.org) platform to identify the relationships between the alterations of hub genes and survivals of PA patients^39^, Kaplan-Meier curves were generated from the PanCancer Atlas of TCGA database. The following settings of genomic profiles were used for the analysis: “mutations”, “putative copy number alterations from GISTIC”, and “mRNA expression z-scores relative to diploid samples (RNA-seq V2 RSEM)”. Complete samples (168 PA patients/cases) were selected for survival analyses, which included OS, DFS, progression-free survival (PFS), and disease-specific survival (DSS) analyses. Next, using univariate Cox proportional hazard analyses, risk factors were considered to be statistically significant with a *P*-value < 0.05, which was further analyzed by multivariate analysis to avoid confounding effects between each risk factor in the TIMER database^37^.

### Diagnostic analysis of hub genes

To verify the mRNA expression level of the selected hub genes in pancreatic tissues, related published online data were obtained from TCGA and Genotype-Tissue Expression (GTEx) project datasets and analyzed using the GEPIA platform^38^. The mRNA microarray datasets were downloaded from the online Oncomine database (https://www.oncomine.org) to evaluate the diagnostic ability of hub gene expressions in tissues for PA. Circulating proteins encoded by these hub genes in blood were screened by the Human Protein Atlas (HPA) platform (https://www.proteinatlas.org/) and further verified for diagnostic value using ELISAs. To determine the diagnostic efficacies of hub genes for PA, MedCalc (version 18.2.1) was used to generate a receiver operating characteristic (ROC) curve and calculate the sensitivity and specificity of the area under the ROC curve (AUC). Hub genes that met the criteria (AUC > 0.5 and *P*-value < 0.05) were identified as potential diagnostic biomarkers.

### Cell lines and Western blot analysis

Human PA cell lines (BxPC-3, CFPAC-1, and MIA PaCa-2) and human normal pancreatic cells (HPDE) were purchased from the Chinese Academy of Sciences Cell Bank (Shanghai, China). Cells were lysed using RIPA buffer (Solarbio Life Science, Beijing, China) containing 1% protease inhibitors, and protein was quantified using a NanoDrop ND-1000 Spectrophotometer (Thermo Fisher Scientific, Waltham, MA, USA). Total protein (30 µg) was separated using 15% SDS-PAGE and transferred to polyvinylidene difluoride membranes (Roche, Basel, Switzerland). After blocking with 3% bovine serum albumin for 1 h, the membranes were incubated with the following primary antibodies: mouse anti-DLGAP5 (#sc-377004, 1:500; Santa Cruz Biotechnology, Santa Cruz, CA, USA), mouse anti-KPNA2 (#sc-55538, 1:500; Santa Cruz Biotechnology), mouse anti-HLA-A (#sc-365485, 1:500; Santa Cruz Biotechnology), mouse anti-ISG15 (#sc-166755, 1:500; Santa Cruz Biotechnology), and rabbit anti-IFI27 (#ER60299, 1:500; Hangzhou HuaAn Biotechnology, Hangzhou, China) overnight at 4 °C. For verification of equal protein loading, mouse monoclonal IgG anti-β-actin (#sc-47778, 1:500; Santa Cruz Biotechnology) was used as a loading control. After extensive washes, the membranes were incubated with secondary antibodies [(m-IgGκ BP-HRP, 1:4,000; Santa Cruz Biotechnology) or (anti-rabbit IgG, horseradish peroxidase-linked antibody, 1:4,000; Cell Signaling Technology, Danvers, MA, USA)] for 1 h at room temperature. Signals were visualized using an Immobilon Western Chemiluminescent HRP substrate (Millipore, Burlington, MA, USA). All bands and relative band intensities were analyzed and quantified by ImageJ software (National Institutes of Health, Bethesda, MD, USA). The relative band intensity was expressed as the ratio of expressed protein to the loading control (β-actin). The experiments were independently repeated 3 times, and data are presented as the mean ± standard deviation from a representative experiment.

### Tissue microarray (TMA) and immunohistochemical analysis of the IFN-stimulated gene 15 (ISG15) protein

The TMA contains well-documented clinicopathological information, all of which was obtained with patient informed consent. The use of clinical samples was approved by the local Ethics Committee. TMA and ethical approval documentation were provided by Shanghai Outdo Biotech (Shanghai, China). The expression of ISG15 was analyzed using a TMA, which contained 35 cases of primary pancreatic ductal adenocarcinoma (PDAC) tissues and 22 cases of matched adjacent noncancerous tissues with 1 point for each tissue. Standard immunohistochemistry (IHC) procedures were performed on the TMA slide using ISG15 antibody (#sc-166755, 1:100; Santa Cruz Biotechnology). The staining intensity scores were no staining (score = 0), light brown (score = 1), brown (score = 2), and dark brown (score = 3), while the percentage scores of positive cells were determined as < 10% (score = 0), 10%–25% (score = 1), 26%–50% (score = 2), 51%–75% (score = 3), and > 75% (score = 4)^40^. The IHC_ISG15_ score was determined by multiplying the intensity score and the percentage score. IHC results were independently evaluated by 2 pathologists blinded to the clinical information. IHC_ISG15_ scores ≥ 6 in cancer tissues were defined as a “high expression level of ISG15.”

### TMA and immunohistochemical analyses of disks large-associated protein 5 (DLGAP5)

The details are presented in the supplementary materials and methods.

### Validation of the diagnostic value of serum ISG15

PA patients were enrolled at the Department of Pancreatic Cancer of Tianjin Medical University Cancer Institute and Hospital from December 2019 to May 2020. Healthy controls were recruited at the Cancer Prevention Center of Tianjin Medical University Cancer Institute and Hospital. Participants with no evidence of pancreatic disease or other abnormalities based on the results of wellness check-ups including laboratory and radiological findings were enrolled as healthy controls. This study was approved by the Ethics Committee of Tianjin Medical University Cancer Institute and Hospital (bc2019104), and written informed consent was obtained from all PA patients and healthy controls.

A total of 78 serum samples were obtained from 40 PA patients and 38 healthy controls. The inclusion criteria were as follows: patients with histologically confirmed primary PA, PA patients admitted to the hospital for the first time without treatment, and PA patients and healthy controls aged > 18 years who did not have common viral infections. The exclusion criteria were as follows: PA patients or healthy controls taking immunotherapeutic drugs or antiviral drugs, PA patients or healthy controls with immune diseases, patients with both PA and other cancers, and healthy controls with a history of pancreatic disease.

The peripheral blood samples were centrifuged at 4 °C for 15 min at 3,000 rpm, and supernatant serum was stored at −80 °C until analysis. Serum ISG15 levels were analyzed according to the manufacturer’s instructions using an ELISA kit (CSB-E12075h; Cusabio, Houston, TX, USA). The details are presented in the supplementary materials and methods.

### Statistical analysis

Statistical analyses were performed using SPSS statistical software for Windows, version 25.0 (IBM, Armonk, NY, USA) and Prism 8.0 (GraphPad Software, San Diego, CA, USA). All quantitative data are expressed as the means and standard deviations from 2 or 3 separate experiments. For western blot analysis, statistical comparison between the 2 groups (PA cell *vs*. HPDE cell groups) was analyzed using Student’s *t*-tests. For IHC analysis, categorical variables were analyzed using the chi-square test. The Kaplan-Meier method and log-rank tests were used to estimate the survival times of PA patients. For ELISAs, differences between the PA group and the healthy control group were assessed using the Mann-Whitney U test (continuous variables and nonparametric analyses). ROC curves were established to assess sensitivity, specificity, and AUCs with 95% confidence intervals (CIs). All *P*-values were 2-sided, and *P* < 0.05 was considered statistically significant.

## Results

### Identification of DEGs in PA

**Figure 1:**
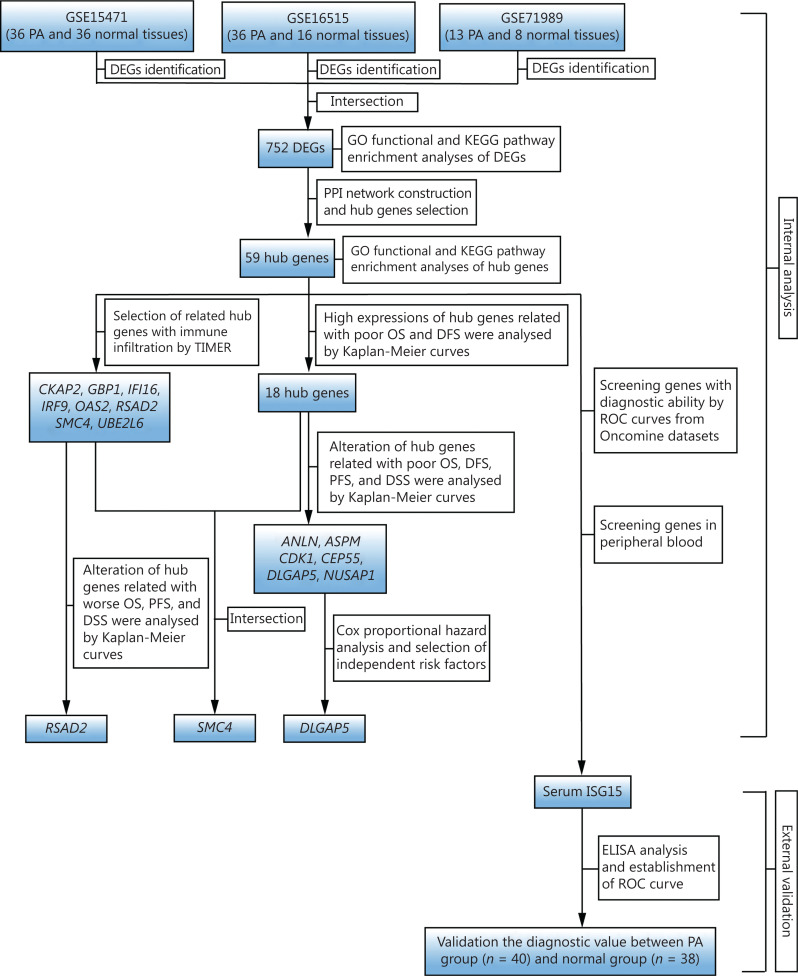
Flow chart of the current study. PA, pancreatic adenocarcinoma; DEGs, differentially expressed genes; GO, Gene Ontology; KEGG, Kyoto Encyclopedia of Genes and Genomes; PPI, protein-protein interaction; TIMER, Tumor Immune Estimation Resource; OS, overall survival; DFS, disease-free survival; PFS, progression-free survival; DSS, disease-specific survival; ELISA, enzyme-linked immunosorbent assay; ROC, receiver operating characteristic; *n*, number.

To illustrate our study protocol, a flow chart is presented in **Figure 1**. The DEGs (1,815 in GSE15471, 1,775 in GSE16515, and 4,007 in GSE71989) were extracted from standardized microarray analyses. Three DEG datasets were then uploaded to a Venn diagram web-based tool (http://bioinformatics.psb.ugent.be/webtools/Venn/) to obtain the intersection set, which included 752 DEGs between PA tissues and noncancerous tissues (**Supplementary Figure S1**), with 621 upregulated genes and 131 downregulated genes.

### GO functional and KEGG pathway enrichment analyses of 752 DEGs

**Figure 2:**
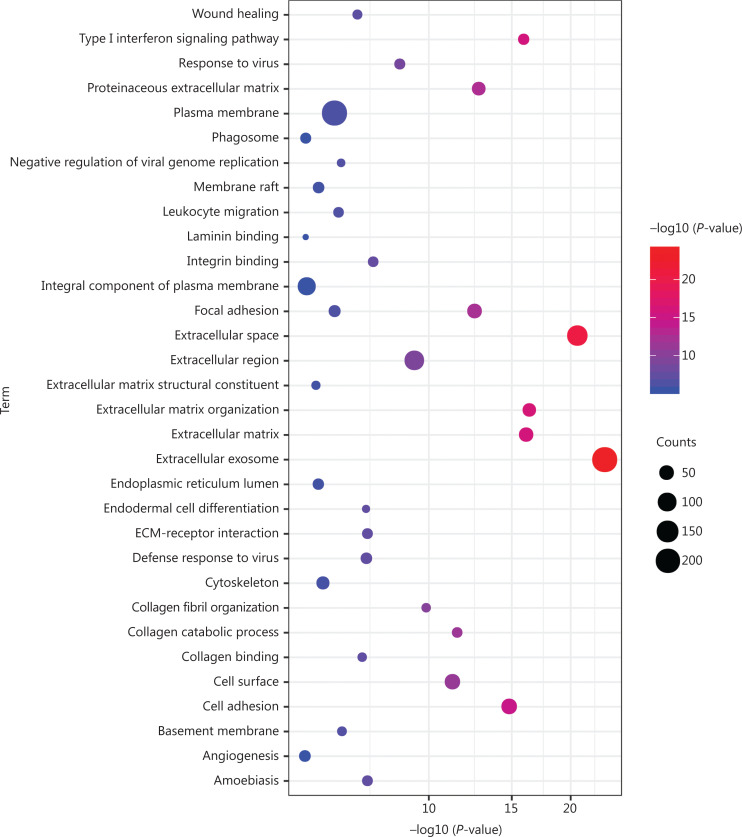
GO functional and KEGG pathway enrichment analyses of 752 DEGs. GO functional and KEGG pathway enrichment analyses of these DEGs are shown in a bubble chart. The y-axis labels show enriched GO functional and KEGG pathway terms of these DEGs, and the x-axis labels show the –log 10 (*P*-values) of the significantly enriched terms. Count represents the number of DEGs enriched in 1 GO functional or KEGG pathway term. GO, Gene Ontology; KEGG, Kyoto Encyclopedia of Genes and Genomes; DEGs, differentially expressed genes; ECM, extracellular matrix.

As shown in **Figure 2 and Supplementary Table S1**, GO analysis revealed that 752 DEGs were significantly enriched in BPs, mainly including extracellular matrix (ECM) organization, type I IFN signaling pathway, cell adhesion, and collagen catabolic process. For MF, these DEGs were significantly enriched in integrin binding, collagen binding, ECM structural constituent, and laminin binding. For CC, the extracellular exosome, extracellular space, ECM, and proteinaceous ECM were significantly enriched. KEGG pathway analysis showed that these DEGs were significantly enriched in ECM-receptor interaction, amoebiasis, focal adhesion, and phagosome.

### Visualized PPI network construction and module analysis

**Figure 3:**
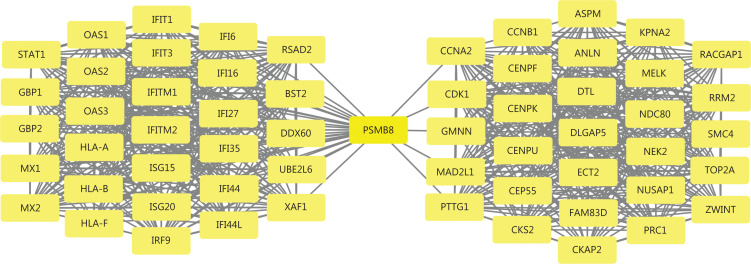
The most densely connected module from the PPI network of 752 DEGs. This module had 59 nodes and 794 edges. All DEGs (59 genes) from this module, which were considered hub genes, had node degrees ≥ 20. The average node degree = 26.9. PPI, protein-protein interaction; DEGs, differentially expressed genes.

**Figure 4:**
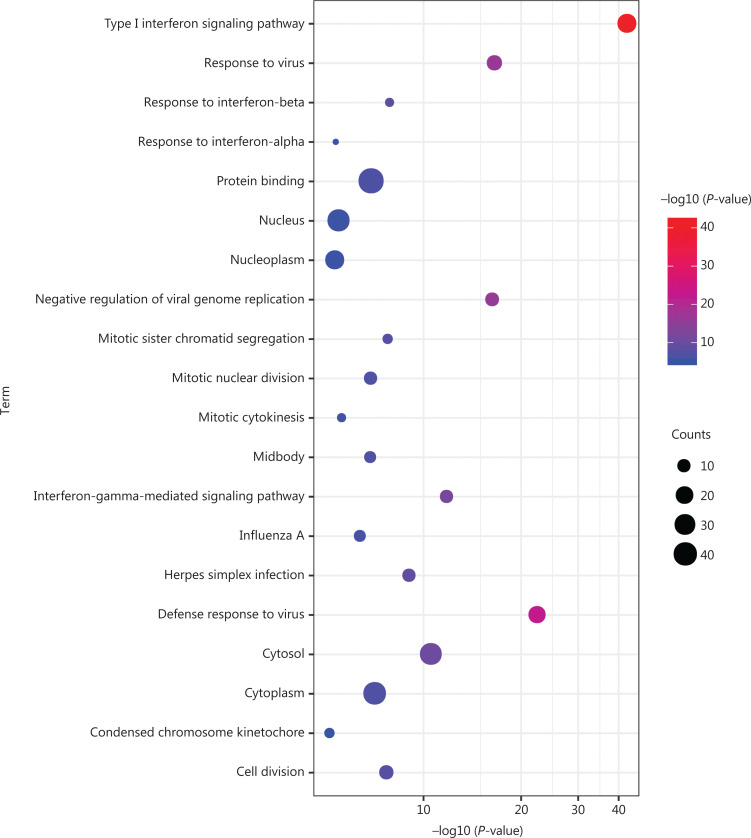
GO functional and KEGG pathway enrichment analyses of 59 hub genes. GO functional and KEGG pathway enrichment analyses of hub genes are shown in a bubble chart. The y-axis labels show enriched GO functional and KEGG pathway terms of hub genes, and the x-axis labels show the –log 10 (*P*-values) of the significantly enriched terms. Count represents the number of hub genes enriched in 1 GO functional or KEGG pathway term. GO, Gene Ontology; KEGG, Kyoto Encyclopedia of Genes and Genomes.

The visualized PPI network of 752 DEGs was generated and displayed using Cytoscape (**Supplementary Figure S2**). The most densely connected module was extracted from this PPI network (**Figure 3 and Supplementary Figure S2**). A total of 59 genes in this module were identified as hub genes with degrees ≥ 20. Geminin (*GMNN*) was downregulated, and other hub genes were upregulated. These genes were mainly enriched in the defense response to virus, response to virus, mitotic sister chromatid segregation, cell division, mitotic nuclear division, and type I IFN signaling pathways (**Figure 4 and Supplementary Table S2**). Two significantly enriched pathways in KEGG were herpes simplex infection and influenza A (**Figure 4 and Supplementary Table S2**).

### The Hub genes related to immune cell infiltration were selected

**Figure 5:**
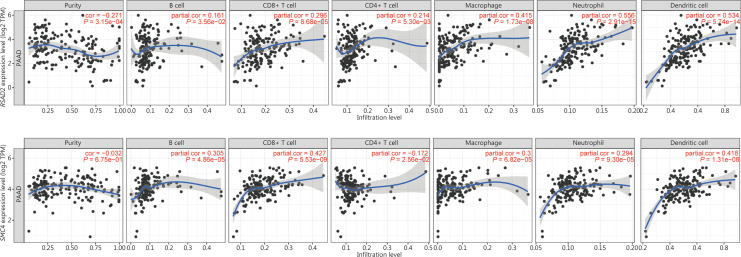
*RSAD2* and *SMC4* were related to immune cell infiltration. *RSAD2* and *SMC4* were significantly correlated with infiltrating levels of B cells, CD4+ T cells, CD8+ T cells, macrophages, neutrophils, and dendritic cells. Cor, correlation.

We speculated that the results of GO and KEGG enrichment analyses of these 59 hub genes were related to the regulation of the immune system. It was therefore necessary to further understand the association of hub genes and immune cell infiltration in the PA microenvironment. Scatterplots and fitting curves of the correlations between hub genes and immune cell infiltration were generated by TIMER (**Figure 5 and Supplementary Table S3**). The results suggested that the expressions of cytoskeleton associated protein 2 (*CKAP2*), guanylate binding protein 1 (*GBP1*), IFN gamma inducible protein 16 (*IFI16*), IFN regulatory factor 9 (*IRF9*), 2′-5′-oligoadenylate synthetase 2 (*OAS2*), radical S-adenosyl methionine domain containing 2 (*RSAD2*), structural maintenance of chromosome 4 (*SMC4*), and ubiquitin conjugating enzyme E2 L6 (*UBE2L6*) had significant correlations with the infiltration levels of all 6 immune cells (B cells, CD4+ T cells, CD8+ T cells, macrophages, neutrophils, and dendritic cells) (*P*-value < 0.05). Other hub genes were correlated with some of the above infiltrating immune cells. Additionally, *RSAD2* was more closely associated with macrophages, neutrophils, and dendritic cells than with other infiltrating immune cells (partial correlation > 0.3 and *P* < 0.05) (**Figure 5 and Supplementary Table S3**), while alterations in *RSAD2* were correlated with poor OS, PFS, and DSS of PA patients (**Supplementary Figure S10**). *SMC4* was more closely associated with B cells, CD8+ T cells, and dendritic cells than with other infiltrating immune cells (partial correlation > 0.3 and *P* < 0.05) (**Figure 5 and Supplementary Table S3**), while high expression of *SMC4* was correlated with worse OS and DFS of PA patients (**Supplementary Figure S3 and Figure S4**).

### Prognostic capability evaluation of hub genes

As shown in **Supplementary Figure S3 and Figure S4**, overexpression of anillin actin binding protein (*ANLN*), abnormal spindle microtubule assembly (*ASPM*), cyclin A2 (*CCNA2*), cyclin B1 (*CCNB1*), cyclin dependent kinase 1 (*CDK1*), centrosomal protein 55 (*CEP55*), CDC28 protein kinase regulatory subunit 2 (*CKS2*), *DLGAP5*, family with sequence similarity 83 member D (*FAM83D*), maternal embryonic leucine zipper kinase (*MELK*), nucleolar and spindle associated protein 1 (*NUSAP1*), 2′-5′-oligoadenylate synthetase 3 (*OAS3*), protein regulator of cytokinesis 1 (*PRC1*), pituitary tumor-transforming 1 (*PTTG1*), Rac GTPase activating protein 1 (*RACGAP1*), ribonucleotide reductase regulatory subunit M2 (*RRM2*), *SMC4*, and topoisomerase II alpha (*TOP2A*) were correlated with both poor OS and DFS among PA patients using the Kaplan-Meier curve on the online GEPIA platform (log-rank *P*-value < 0.05). We screened genes for their relationships to OS and DFS and further determined the relationships between changes in these genes and survival using the online cBioPortal platform. As shown in **Supplementary Figure S5**, alterations in *ANLN*, *ASPM*, *CDK1*, *CEP55*, *DLGAP5*, and *NUSAP1* were related to OS, DFS, PFS, and DSS in the PanCancer Atlas of TCGA database (log-rank *P*-value < 0.05). Alterations in these genes included missense mutations, truncating mutations, amplifications, and high mRNA expressions. Thus, high expressions and alterations of *ANLN*, *ASPM*, *CDK1*, *CEP55*, *DLGAP5*, and *NUSAP1* were considered potential prognostic biomarkers of PA. Cox proportional hazard analysis was then performed using the online TIMER platform (**Table 1**). The results of univariate analysis showed that age, tumor purity, CD8+ T cell infiltration, and 6 hub genes were risk factors for survival. The results of multivariate analyses showed that the age of patients and *DLGAP5* mRNA expression might be independent risk factors for survival [age: hazard ratio (HR): 1.031, 95% CI: 1.009–1.053, *P*-value = 0.006; *DLGAP5*: HR: 0.594, 95% CI: 0.359–0.983, *P*-value = 0.043), as shown in **Table 1**.

**Table 1:** Univariate and multivariate Cox regression analyses for survival, using the TIMER database

Variables	Univariate analysis			Multivariate analysis		
	HR	95% CI	*P*-value	HR	95% CI	*P*-value
Age	1.028	1.007–1.049	0.010*	1.031	1.009–1.053	0.006*
Gender, male	0.813	0.541–1.222	0.319	–	–	–
Race						
Black	1.210	0.324–4.519	0.777	–	–	–
White	1.264	0.511–3.127	0.613	–	–	–
Stage						
2	2.350	1.078–5.125	0.032*	–	–	–
3	1.055	0.129–8.643	0.960	–	–	–
4	1.594	0.327–7.780	0.564	–	–	–
Tumor purity	0.454	0.214–0.963	0.040*	0.687	0.288–1.637	0.396
B cells	1.664	0.112–24.800	0.712	–	–	–
CD8+ T cells	18.719	1.056–331.683	0.046*	6.825	0.184–253.697	0.298
CD4+ T cells	0.010	0.000–1.822	0.083	–	–	–
Macrophages	0.861	0.026–28.261	0.933	–	–	–
Neutrophils	476.588	0.286–795522.500	0.103	–	–	–
Dendritic cells	2.292	0.444–11.841	0.322	–	–	–
*ANLN*	1.573	1.297–1.908	< 0.001*	1.323	0.879–1.991	0.179
*ASPM*	1.749	1.322–2.312	< 0.001*	0.670	0.366–1.228	0.195
*CDK1*	1.746	1.354–2.252	< 0.001*	1.041	0.514–2.108	0.912
*CEP55*	1.765	1.387–2.246	< 0.001*	1.626	0.812–3.253	0.170
*DLGAP5*	1.745	1.367–2.227	< 0.001*	0.594	0.359–0.983	0.043*
*NUSAP1*	1.769	1.358–2.305	< 0.001*	1.061	0.550–2.044	0.861

References included: female for gender, white for race, and stage 1 for tumor stage. *P < 0.05 was considered statistically significant. TIMER, Tumor Immune Estimation Resource; HR, hazard ratio; CI, confidence interval.

### Diagnostic capability evaluation of hub genes

**Figure 6:**
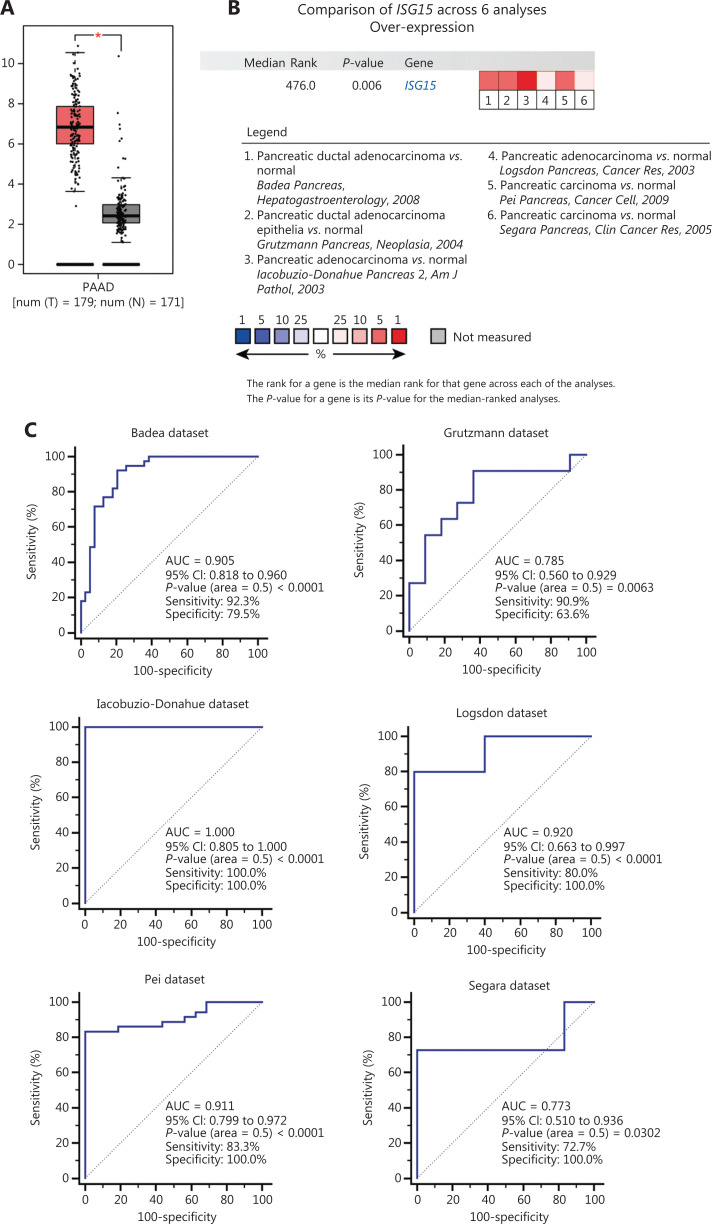
Analyses of *ISG15* mRNA expression in PA patients *vs.* normal controls for diagnosis. (A) Analysis of *ISG15* mRNA expression in PA *vs.* normal pancreatic tissues from TCGA and GTEx datasets. The following settings were used for the analysis: “expression on box plots”, “gene = *ISG15*”, “|log_2_(FC)| cut-off = 1”, “*P*-value cut-off = 0.01”, “datasets = PAAD”, “log scale = log_2_(TPM + 1)”, “jitter size = 0.4”, and “matched TCGA normal and GTEx data”. (B) Heat map of *ISG15* mRNA expression in PA *vs.* normal tissues with a *P*-value < 0.05 from 6 independent datasets from Oncomine. The 6 datasets included the Badea dataset^29^, Grutzmann dataset^41^, Iacobuzio-Donahue dataset^42^, Logsdon dataset^43^, Pei dataset^30^, and Segara dataset^44^. (C) ROC curve analysis of *ISG15* mRNA expression with a *P*-value < 0.05 was used to diagnose PA from the 6 independent datasets from Oncomine. PA, pancreatic adenocarcinoma; T, tumor; N, normal; *P-value < 0.01; TCGA, The Cancer Genome Atlas; GTEx, Genotype-Tissue Expression; FC, fold change; PAAD, pancreatic adenocarcinoma; TPM, transcripts per million; ROC, receiver operating characteristic; AUC, area under the ROC curve; CI, confidence interval.

To assess the mRNA expressions of 59 individual hub genes in PA and normal pancreatic tissues, we analyzed 8 datasets from Oncomine and found that the differential mRNA expression levels of the major histocompatibility complex, class I, A (*HLA-A*), IFN alpha inducible protein 27 (*IFI27*), *ISG15*, and karyopherin subunit alpha 2 (*KPNA2*) approximately correlated with the top 1% of genes in gene rank in the 3 datasets (**Supplementary Figure S6**). High mRNA expression levels of *HLA-A*, *IFI27*, *ISG15*, and *KPNA2* in PA tissues *vs*. normal pancreatic tissues were considered statistically significant (*P* < 0.05) in 5 or 6 datasets (**Supplementary Table S4**). Next, 350 samples from TCGA and GTEx databases were used to analyze the mRNA expression levels of *ISG15* in PA *vs*. normal tissues. The overexpression of *ISG15* in PA tissues was significantly higher than that in normal tissues (*P* < 0.05) (**Figure 6A**), which confirmed the results of the analysis from Oncomine. A total of 201 samples from 6 Oncomine datasets were used to evaluate the diagnostic value of *ISG15* mRNA for PA by ROC curve analysis [AUC = 0.905 with 92.3% sensitivity and 79.5% specificity in the Badea dataset (*P* < 0.0001)^29^; AUC = 0.785 with 90.9% sensitivity and 63.6% specificity in the Grutzmann dataset (*P* = 0.0063)^41^; AUC = 1.000 with 100% sensitivity and 100% specificity in the Iacobuzio-Donahue dataset (*P* < 0.0001)^42^; AUC = 0.920 with 80.0% sensitivity and 100% specificity in the Logsdon dataset (*P* < 0.0001)^43^; AUC = 0.911 with 83.3% sensitivity and 100% specificity in the Pei dataset (*P* < 0.0001)^30^; and AUC = 0.773 with 72.7% sensitivity and 100% specificity in the Segara dataset (*P* = 0.0302)^44^] (**Figure 6B and 6C**). Similarly, we evaluated the diagnostic value of *HLA-A*, *IFI27*, and *KPNA2* mRNA expressions in the same way (**Supplementary Figure S7, Figure S8, and Figure S9**).

### External validation of the protein levels of the hub genes

**Figure 7:**
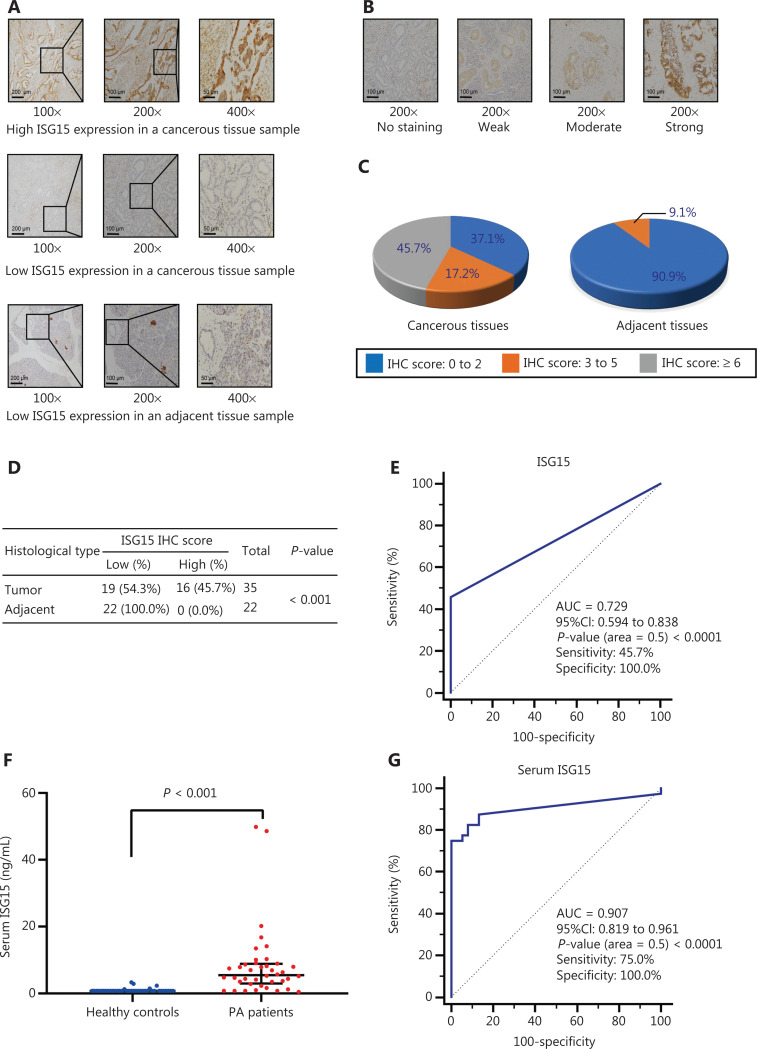
High protein levels of ISG15 in cancerous tissues and serum of PA patients. (A) Protein expressions of ISG15 in cancerous and adjacent noncancerous tissues based on the IHC score (staining percentage × intensity). Typical images of IHC staining for ISG15 were magnified 100-fold, 200-fold, and 400-fold in the TMA (scale bars: 200 μm, 100 μm, and 50 μm from left to right). ISG15 expression was classified as follows: samples with IHC scores < 6 were defined as low expression, and scores ≥ 6 were defined as high expression. The percentage of cells staining positive was scored as follows: < 10% (score = 0), 10%–25% (score = 1), 26%–50% (score = 2), 51%–75% (score = 3), and 76%–100% (score = 4). (B) Representative images of PDAC tissues with no staining/weak/moderate/strong ISG15 expression magnified 200-fold. Scale bars: 100 μm. The staining intensity was scored as follows: no staining (score = 0), weak (score = 1), moderate (score = 2), and strong (score = 3). (C) The proportion of cancerous (**Figure 7C**, left) and adjacent noncancerous (**Figure 7C**, right) tissues with ISG15 protein levels based on IHC scores (0 to 12). (D) Correlation between the protein level of ISG15 and histological type. IHC scores ≥ 6 were considered high levels of ISG15. A *P*-value < 0.05 was considered to be statistically significant. (E) An ROC curve of ISG15 protein levels [high levels of ISG15 (IHC scores ≥ 6) and low levels of ISG15 (IHC score < 6)] was used to differentiate cancerous tissues (*n* = 35) from adjacent noncancerous tissues (*n* = 22) (*P* < 0.001). (F) High protein levels of serum ISG15 in PA patients (*n* = 40) compared with those in healthy controls (*n* = 38) (*P* < 0.001). (G) A ROC curve of the serum ISG15 levels was used to differentiate PA patients from healthy controls. PDAC, pancreatic ductal adenocarcinoma; IHC, immunohistochemistry; TMA, tissue microarray; *n*, number, ROC, receiver operating characteristic; AUC, area under the ROC curve; CI, confidence interval.

We then measured the protein levels of the hub genes that may have potential clinical value. We detected the protein levels of DLGAP5, KPNA2, HLA-A, ISG15, and IFI27 in 3 kinds of PA cells (BxPC-3, CFPAC-1, and MIA PaCa-2) and normal pancreatic cells (HPDE) by Western blot (**Supplementary Figure S11A**). The protein levels of DLGAP5, KPNA2, and IFI27 in 2 kinds of PA cells (BxPC-3 and MIA PaCa-2) were higher than those in normal pancreatic cells (**Supplementary Figure S11B**). The protein levels of HLA-A and ISG15 in 3 kinds of PA cells were higher than those in normal pancreatic cells (**Supplementary Figure S11B**). Using the HPA platform to determine protein levels for these genes in clinical samples, we observed that only the protein encoded by *ISG15* was expressed in tissues and secreted into the blood. We further detected the protein level of ISG15 in the TMA containing PDAC and normal adjacent pancreatic tissues (**Figure 7A**) and found that staining for ISG15 was strong in PDAC tumor cells (**Figure 7A**, top), whereas in noncancerous tissues, the staining was weak or undetectable, except in islet cells that showed positive staining (**Figure 7A**, bottom). The levels of ISG15 protein in cancerous tissues were significantly higher than those in adjacent noncancerous tissues (**Figure 7A**, **7C**, **7D**). In all PDAC tissues, the number of cases with high levels of ISG15 with IHC scores greater than or equal to 6 accounted for 45.7% (**Figure 7C**, left), the number of cases with scores between 3 and 5 accounted for 17.2% (**Figure 7C**, left), and the number of cases with scores between 0 and 2 accounted for 37.1% (**Figure 7C**, left). In all adjacent noncancerous tissues, 100.0% exhibited low levels (IHC scores less than 6, **Figure 7D**), in which the number of cases with IHC scores between 0 and 2 accounted for 90.9% (**Figure 7C**, right), and those between 3 and 5 accounted for 9.1% (**Figure 7C**, right). However, the ISG15 protein level showed no significant correlation with other clinicopathological factors including age, gender, pathological grade, T (tumor) stage, N (node) stage, and M (metastasis) stage (**Supplementary Table S5**). The ISG15 protein levels were evaluated to differentiate cancerous tissues from adjacent noncancerous tissues using ROC curve analysis (AUC: 0.729, 95% CI: 0.594–0.838, sensitivity: 45.7%, specificity: 100.0%) (**Figure 7E**). Next, we validated the protein levels of serum ISG15 in PA patients and healthy controls using ELISAs. Thirty-eight healthy participants were included as healthy controls (16 males and 22 females), and 40 PA patients (16 males and 24 females) were included in this study. The age range of PA patients was 36 years to 75 years, and the age range of healthy controls was 29 years to 57 years. Significant differences in serum ISG15 levels were found between the 2 groups (*P* < 0.001) (**Figure 7F**). ROC curve analysis was performed to differentiate PA from healthy controls (AUC: 0.907, 95% CI: 0.819–0.961, sensitivity: 75.0%, specificity: 100.0%) (**Figure 7G**). The results showed that the ISG15 protein could be a potential diagnostic biomarker of PA in both serum and tissues.

## Discussion

In our study, the majority of 752 DEGs were enriched in the ECM-receptor interaction pathway. It is well-known that the ECM is one of the unique characteristics of PA. Anomalous proliferation and deposition of the ECM promote the formation of the microenvironment in PA, thus facilitating tumor progression, metastasis, and therapeutic resistance^45,46^. In addition, we found that the most densely connected module from the PPI network consisted of 59 hub genes with degrees ≥ 20. The BPs of hub genes were mainly enriched in the type I IFN signaling and defense response to virus pathways, and the KEGG pathway was mainly enriched in herpes simplex infection and influenza A, suggesting that some hub genes may play an important role in the defense response to virus through the type I IFN signaling pathway. The IFN response and antiviral defense mechanisms within tumors can restrict viral replication, which is a challenge for OVs^47^. Blackham et al.^48^ reported that intact IFN-mediated defenses were the main causes of OV resistance in PA cells. Thus, tumor cells that use OV selective targeting generally have defective IFN systems. Some researchers have found low expression of some genes induced by type I IFN in tumor cells susceptible to OVs, and downregulation of these genes in OV-resistant tumor cells increased the sensitivity of the cells to OV infection^13,49,50^. These genes may be used as potential biomarkers to predict the success of OV infection and replication^13^.

In the present study, we determined the associations of hub genes and immune cell infiltration in the microenvironment of PA using the online TIMER platform. We found for the first time that 8 hub genes (*CKAP2*, *GBP1*, *IFI16*, *IRF9*, *OAS2*, *RSAD2*, *SMC4*, and *UBE2L6*) were related to immune cell infiltration. GBP1^51^, IFI16^52^, IRF9^51^, *OAS2*^47^, and RSAD2^53^ were demonstrated to be restriction factors in some OV infections or replications. Because these genes were mainly enriched in the defense response to viruses (**Supplementary Table S2**), we speculated that high expression levels of these genes may have led to resistance of PA cells to OV treatments, which requires confirmation in future studies. RSAD2 is an ISG that is a broadly acting effector of the type I/II IFN antiviral response against several enveloped viruses, such as herpes virus, influenza A virus, and measles virus (MV)^53–55^. Kurokawa et al.^53^ reported that RSAD2 was a restriction factor of the oncolytic MV that inhibited the release of MV in infected ovarian cancer cells (SR-B2). Human glioblastoma cells were infected by attenuated MV as a kind of oncolytic virotherapy, which activated *RSAD2* expression in tumor cells^47^. They suggested that *RSAD2* activation could be a predictive biomarker for the response to oncolytic MV therapy. However, the role of *RSAD2* in OV therapy for PA remains unclear and should be further investigated in future studies. The structural maintenance of chromosome 4 protein is encoded by *SMC4*, which is a structural maintenance protein of the chromosome (SMC) family member. High expression of SMC4 is related to tumor size, dedifferentiation, advanced stages, and vascular invasion of liver cancer^56^. In glioma and colorectal cancer, upregulated expression of SMC4 promoted the tumor cell growth rate, migration and invasion^57–59^. Overexpression of SMC4 is an independent prognostic factor in lung adenocarcinoma^60^. High expression of *SMC4* predicted worse prognosis of pancreatic ductal adenocarcinoma patients^61^. In the present study, we first observed that *SMC4* was associated with immune cell infiltration in the microenvironment of PA. Moreover, *SMC4* was correlated with the OS and DFS of PA patients. Nevertheless, the role of *SMC4* in the immune microenvironment of PA remains unclear and should be further investigated in future studies.

Our results indicated that alterations in *ANLN*, *ASPM*, *CDK1*, *CEP55*, *DLGAP5*, and *NUSAP1* were significantly related to the survival of PA patients. DLGAP5 is an important mitotic spindle protein, and the upregulation of DLGAP5 contributes to the proliferation, migration, and invasion of multiple cancers^62–64^. Overexpression of the DLGAP5 protein or increased mRNA expression are associated with an unfavorable prognosis in multiple cancers, such as colorectal cancer^64^, ovarian cancer^65^, and lung cancer^66,67^. However, until recently, only 1 study has been performed to identify *DLGAP5* mRNA expression as a potential biomarker for the diagnosis and prognosis of PA by bioinformatics analysis^68^. To the best of our knowledge, there are no reports of detecting DLGAP5 protein levels in PA tissues. In the present study, we found that alterations in *DLGAP5* were related to worse OS, DFS, PFS and DSS in PA patients. More importantly, *DLGAP5* mRNA expression might be an independent risk factor for the prognoses of PA patients, using bioinformatics analyses. To determine whether the protein level of DLGAP5 has prognostic value for PA, we used immunohistochemical methods to detect the protein level of DLGAP5 in the TMA of PA patients with prognostic information. No positively stained cells were found in any adjacent noncancerous tissues (an IHC_DLGAP5_ score of 0 was defined as a negative result), while some cancerous tissues had a small number of positively stained cells (an IHC_DLGAP5_ score of 1 or 2 was defined as a weakly positive result) (**Supplementary Figure S12A, S12B**). Although we found that weakly positive expression of DLGAP5 protein was associated with higher pathological grade and shorter OS of PDAC patients in this study (*P* < 0.05) (**Supplementary Table S6 and Figure S12C**), we believe that whether the protein level of DLAGP5 has prognostic value needs to be further confirmed by increasing the number of clinical specimens. We will collect more clinical samples in the future to verify the protein level of DLGAP5 in PA and normal adjacent pancreatic tissues and to explore its prognostic value. In addition, studies on the regulatory mechanism of DLGAP5 in PA have rarely been reported, which should be conducted and validated in future investigations.

*ISG15* encodes a 15-kDa ubiquitin-like protein, which is induced by IFN-α and IFN-β. KRAS-associated phenotypes of PA cells are reversed by knocking down the ISG15 pathway proteins (free ISG15 and ISG15 conjugates), which by itself or in combination with anti-PD-1 treatment may contribute to improved survival for patients with PA^69^. Another study reported that extracellular ISG15 maintained the stem cell-like features of PA cells, such as clonogenicity, invasiveness, and spheroid formation, by an autocrine mode of action^70^. Using data analysis of the Oncomine database, we showed that *ISG15* mRNA expressions in tissues may have potential value in the diagnosis of PA. In terms of the protein level of ISG15 in clinical samples, little is known about its diagnostic value in PA. In our study, the differential protein levels of ISG15 were measured between PA and adjacent noncancerous tissues by IHC. The protein level of serum ISG15 was further validated by ELISA, and the diagnostic role of serum ISG15 was identified by ROC analysis.

## Conclusions

Using bioinformatics analyses, our study identified *DLGAP5* mRNA expression as a potential independent risk factor for prognoses of PA patients, while showing that *RSAD2* and *SMC4* were related to immune cell infiltration. The protein levels of ISG15 in PA tissues were significantly higher than those in adjacent noncancerous tissues, which were validated by IHC. Additionally, the differential expression of serum ISG15 was validated by ELISAs to show that it could successfully distinguish PA patients from healthy controls by ROC analysis. However, there are several limitations in the current study. First, based on the present data from bioinformatics analyses, functional experiments are needed to verify these results. Moreover, we observed that serum ISG15 was a potential novel non-invasive biomarker for the diagnosis of PA, but this was based on a single cohort with a small sample size. Future studies using larger independent cohorts are needed to validate our findings, and the control group should involve chronic pancreatitis patients.

## References

[1] Elaileh A, Saharia A, Potter L, Baio F, Ghafel A, Abdelrahim M (2019). Promising new treatments for pancreatic cancer in the era of targeted and immune therapies. Am J Cancer Res.

[2] De Angelis R, Sant M, Coleman MP, Francisci S, Baili P, Pierannunzio D (2014). Cancer survival in Europe 1999-2007 by country and age: Results of EUROCARE --5-a population-based study. Lancet Oncol.

[3] Zuckerman DS, Ryan DP (2008). Adjuvant therapy for pancreatic cancer: a review. Cancer.

[4] Groot VP, Rezaee N, Wu W, Cameron JL, Fishman EK, Hruban RH (2018). Patterns, timing, and predictors of recurrence following pancreatectomy for pancreatic ductal adenocarcinoma. Ann Surg.

[5] Kleeff J, Reiser C, Hinz U, Bachmann J, Debus J, Jaeger D (2007). Surgery for recurrent pancreatic ductal adenocarcinoma. Ann Surg.

[6] Mahalingam D, Wilkinson GA, Eng KH, Fields P, Raber P, Moseley JL (2020). Pembrolizumab in combination with the oncolytic virus pelareorep and chemotherapy in patients with advanced pancreatic adenocarcinoma: a Phase Ib study. Clin Cancer Res.

[7] Jiang J, Zhou H, Ni C, Hu X, Mou Y, Huang D (2019). Immunotherapy in pancreatic cancer: new hope or mission impossible?. Cancer Lett.

[8] Ibrahim AM, Wang YH (2016). Viro-immune therapy: a new strategy for treatment of pancreatic cancer. World J Gastroenterol.

[9] Wu J, Cai J (2021). Dilemma and challenge of immunotherapy for pancreatic cancer. Dig Dis Sci.

[10] Andtbacka RH, Kaufman HL, Collichio F, Amatruda T, Senzer N, Chesney J (2015). Talimogene laherparepvec improves durable response rate in patients with advanced melanoma. J Clin Oncol.

[11] Eissa IR, Bustos-Villalobos I, Ichinose T, Matsumura S, Naoe Y, Miyajima N (2018). The current status and future prospects of oncolytic viruses in clinical trials against melanoma, glioma, pancreatic, and breast cancers. Cancers (Basel).

[12] Lawler SE, Speranza MC, Cho CF, Chiocca EA (2017). Oncolytic viruses in cancer treatment: a review. JAMA Oncol.

[13] Hastie E, Cataldi M, Moerdyk-Schauwecker MJ, Felt SA, Steuerwald N, Grdzelishvili VZ (2016). Novel biomarkers of resistance of pancreatic cancer cells to oncolytic vesicular stomatitis virus. Oncotarget.

[14] Moerdyk-Schauwecker M, Shah NR, Murphy AM, Hastie E, Mukherjee P, Grdzelishvili VZ (2013). Resistance of pancreatic cancer cells to oncolytic vesicular stomatitis virus: Role of type I interferon signaling. Virology.

[15] Malilas W, Koh SS, Kim S, Srisuttee R, Cho IR, Moon J (2013). Cancer upregulated gene 2, a novel oncogene, enhances migration and drug resistance of colon cancer cells via STAT1 activation. Int J Oncol.

[16] Yu L, Chen S, Bao H, Zhang W, Liao M, Liang Q (2018). The role of lncRNA CASC2 on prognosis of malignant tumors: a meta-analysis and bioinformatics. Onco Targets Ther.

[17] Pan Z, Li L, Fang Q, Zhang Y, Hu X, Qian Y (2018). Analysis of dynamic molecular networks for pancreatic ductal adenocarcinoma progression. Cancer Cell Int.

[18] Liu P, Weng Y, Sui Z, Wu Y, Meng X, Wu M (2016). Quantitative secretomic analysis of pancreatic cancer cells in serum-containing conditioned medium. Sci Rep.

[19] Zhao X, Wang X, Fang L, Lan C, Zheng X, Wang Y (2017). A combinatorial strategy using YAP and pan-RAF inhibitors for treating KRAS-mutant pancreatic cancer. Cancer Lett.

[20] Strnadel J, Choi S, Fujimura K, Wang H, Zhang W, Wyse M (2017). eIF5A-PEAK1 signaling regulates YAP1/TAZ protein expression and pancreatic cancer cell growth. Cancer Res.

[21] Mello SS, Valente LJ, Raj N, Seoane JA, Flowers BM, McClendon J (2017). A p53 super-tumor suppressor reveals a tumor suppressive p53-Ptpn14-Yap axis in pancreatic cancer. Cancer Cell.

[22] Haddad D, Socci N, Chen CH, Chen NG, Zhang Q, Carpenter SG (2016). Molecular network, pathway, and functional analysis of time-dependent gene changes associated with pancreatic cancer susceptibility to oncolytic vaccinia virotherapy. Mol Ther Oncolytics.

[23] Nagashima T, Yamaguchi K, Urakami K, Shimoda Y, Ohnami S, Ohshima K (2020). Japanese version of the cancer genome atlas, JCGA, established using fresh frozen tumors obtained from 5143 cancer patients. Cancer Sci.

[24] Grützmann R, Boriss H, Ammerpohl O, Luttges J, Kalthoff H, Schackert HK (2005). Meta-analysis of microarray data on pancreatic cancer defines a set of commonly dysregulated genes. Oncogene.

[25] Kumar R, Patiyal S, Kumar V, Nagpal G, Raghava GPS (2019). In silico analysis of gene expression change associated with copy number of enhancers in pancreatic adenocarcinoma. Int J Mol Sci.

[26] Yan W, Liu X, Wang Y, Han S, Wang F, Liu X (2020). Identifying drug targets in pancreatic ductal adenocarcinoma through machine learning, analyzing biomolecular networks, and structural modeling. Front Pharmacol.

[27] Mishra NK, Southekal S, Guda C (2019). Survival analysis of multi-omics data identifies potential prognostic markers of pancreatic ductal adenocarcinoma. Front Genet.

[28] Barrett T, Troup DB, Wilhite SE, Ledoux P, Rudnev D, Evangelista C (2007). NCBI GEO: Mining tens of millions of expression profiles--database and tools update. Nucleic Acids Res.

[29] Badea L, Herlea V, Dima SO, Dumitrascu T, Popescu I (2008). Combined gene expression analysis of whole-tissue and microdissected pancreatic ductal adenocarcinoma identifies genes specifically overexpressed in tumor epithelia. Hepatogastroenterology.

[30] Pei H, Li L, Fridley BL, Jenkins GD, Kalari KR, Lingle W (2009). FKBP51 affects cancer cell response to chemotherapy by negatively regulating Akt. Cancer Cell.

[31] Jiang J, Azevedo-Pouly AC, Redis RS, Lee EJ, Gusev Y, Allard D (2016). Globally increased ultraconserved noncoding RNA expression in pancreatic adenocarcinoma. Oncotarget.

[32] The Gene Ontology C (2017). Expansion of the Gene Ontology knowledgebase and resources. Nucleic Acids Res.

[33] Kanehisa M, Furumichi M, Tanabe M, Sato Y, Morishima K (2017). KEGG: New perspectives on genomes, pathways, diseases and drugs. Nucleic Acids Res.

[34] Huang da W, Sherman BT, Lempicki RA (2009). Systematic and integrative analysis of large gene lists using DAVID bioinformatics resources. Nat Protoc.

[35] Szklarczyk D, Morris JH, Cook H, Kuhn M, Wyder S, Simonovic M (2017). The STRING database in 2017: Quality-controlled protein-protein association networks, made broadly accessible. Nucleic Acids Res.

[36] Su G, Morris JH, Demchak B, Bader GD (2014). Biological network exploration with Cytoscape 3. Curr Protoc Bioinformatics.

[37] Li T, Fan J, Wang B, Traugh N, Chen Q, Liu JS (2017). TIMER: a web server for comprehensive analysis of tumor-infiltrating immune cells. Cancer Res.

[38] Tang Z, Li C, Kang B, Gao G, Li C, Zhang Z (2017). GEPIA: a web server for cancer and normal gene expression profiling and interactive analyses. Nucleic Acids Res.

[39] Gao J, Aksoy BA, Dogrusoz U, Dresdner G, Gross B, Sumer SO (2013). Integrative analysis of complex cancer genomics and clinical profiles using the cBioPortal. Sci Signal.

[40] Xie D, Yu S, Li L, Quan M, Gao Y (2020). The FOXM1/ATX signaling contributes to pancreatic cancer development. Am J Transl Res.

[41] Grützmann R, Pilarsky C, Ammerpohl O, Luttges J, Bohme A, Sipos B (2004). Gene expression profiling of microdissected pancreatic ductal carcinomas using high-density DNA microarrays. Neoplasia.

[42] Iacobuzio-Donahue CA, Maitra A, Olsen M, Lowe AW, van Heek NT, Rosty C (2003). Exploration of global gene expression patterns in pancreatic adenocarcinoma using cDNA microarrays. Am J Pathol.

[43] Logsdon CD, Simeone DM, Binkley C, Arumugam T, Greenson JK, Giordano TJ (2003). Molecular profiling of pancreatic adenocarcinoma and chronic pancreatitis identifies multiple genes differentially regulated in pancreatic cancer. Cancer Res.

[44] Segara D, Biankin AV, Kench JG, Langusch CC, Dawson AC, Skalicky DA (2005). Expression of HOXB2, a retinoic acid signaling target in pancreatic cancer and pancreatic intraepithelial neoplasia. Clin Cancer Res.

[45] Neesse A, Bauer CA, Ohlund D, Lauth M, Buchholz M, Michl P (2019). Stromal biology and therapy in pancreatic cancer: ready for clinical translation?. Gut.

[46] Thomas D, Radhakrishnan P (2019). Tumor-stromal crosstalk in pancreatic cancer and tissue fibrosis. Mol Cancer.

[47] Kurokawa C, Iankov ID, Anderson SK, Aderca I, Leontovich AA, Maurer MJ (2018). Constitutive interferon pathway activation in tumors as an efficacy determinant following oncolytic virotherapy. J Natl Cancer Inst.

[48] Blackham AU, Northrup SA, Willingham M, Sirintrapun J, Russell GB, Lyles DS (2014). Molecular determinants of susceptibility to oncolytic vesicular stomatitis virus in pancreatic adenocarcinoma. J Surg Res.

[49] Malilas W, Koh SS, Lee S, Srisuttee R, Cho IR, Moon J (2014). Suppression of autophagic genes sensitizes CUG2-overexpressing A549 human lung cancer cells to oncolytic vesicular stomatitis virus-induced apoptosis. Int J Oncol.

[50] Aref S, Castleton AZ, Bailey K, Burt R, Dey A, Leongamornlert D (2020). Type 1 interferon responses underlie tumor-selective replication of oncolytic measles virus. Mol Ther.

[51] Tarasova IA, Tereshkova AV, Lobas AA, Solovyeva EM, Sidorenko AS, Gorshkov V (2018). Comparative proteomics as a tool for identifying specific alterations within interferon response pathways in human glioblastoma multiforme cells. Oncotarget.

[52] Li G, Cheng J, Mei S, Wu T, Ye T (2018). Tachypleus tridentatus lectin enhances oncolytic vaccinia virus replication to suppress in vivo hepatocellular carcinoma growth. Mar Drugs.

[53] Kurokawa C, Iankov ID, Galanis E (2019). A key anti-viral protein, RSAD2/VIPERIN, restricts the release of measles virus from infected cells. Virus Res.

[54] Wang X, Hinson ER, Cresswell P (2007). The interferon-inducible protein viperin inhibits influenza virus release by perturbing lipid rafts. Cell Host Microbe.

[55] Gizzi AS, Grove TL, Arnold JJ, Jose J, Jangra RK, Garforth SJ (2018). A naturally occurring antiviral ribonucleotide encoded by the human genome. Nature.

[56] Zhou B, Yuan T, Liu M, Liu H, Xie J, Shen Y (2012). Overexpression of the structural maintenance of chromosome 4 protein is associated with tumor de-differentiation, advanced stage and vascular invasion of primary liver cancer. Oncol Rep.

[57] Jiang L, Zhou J, Zhong D, Zhou Y, Zhang W, Wu W (2017). Overexpression of SMC4 activates TGFβ/Smad signaling and promotes aggressive phenotype in glioma cells. Oncogenesis.

[58] Feng XD, Song Q, Li CW, Chen J, Tang HM, Peng ZH (2014). Structural maintenance of chromosomes 4 is a predictor of survival and a novel therapeutic target in colorectal cancer. Asian Pac J Cancer Prev.

[59] Jinushi T, Shibayama Y, Kinoshita I, Oizumi S, Jinushi M, Aota T (2014). Low expression levels of microRNA-124-5p correlated with poor prognosis in colorectal cancer via targeting of SMC4. Cancer Med.

[60] Zhang C, Kuang M, Li M, Feng L, Zhang K, Cheng S (2016). SMC4, which is essentially involved in lung development, is associated with lung adenocarcinoma progression. Sci Rep.

[61] Fukuhisa H, Seki N, Idichi T, Kurahara H, Yamada Y, Toda H (2019). Gene regulation by antitumor miR-130b-5p in pancreatic ductal adenocarcinoma: The clinical significance of oncogenic eps8. J Hum Genet.

[62] Liao W, Liu W, Yuan Q, Liu X, Ou Y, He S (2013). Silencing of DLGAP5 by siRNA significantly inhibits the proliferation and invasion of hepatocellular carcinoma cells. PLoS One.

[63] Zhang X, Pan Y, Fu H, Zhang J (2018). Nucleolar and spindle associated protein 1 (NUSAP1) inhibits cell proliferation and enhances susceptibility to epirubicin in invasive breast cancer cells by regulating cyclin d kinase (CDK1) and DLGAP5 expression. Med Sci Monit.

[64] Branchi V, Garcia SA, Radhakrishnan P, Gyorffy B, Hissa B, Schneider M (2019). Prognostic value of DLGAP5 in colorectal cancer. Int J Colorectal Dis.

[65] Shen J, Yu S, Sun X, Yin M, Fei J, Zhou J (2019). Identification of key biomarkers associated with development and prognosis in patients with ovarian carcinoma: evidence from bioinformatic analysis. J Ovarian Res.

[66] Schneider MA, Christopoulos P, Muley T, Warth A, Klingmueller U, Thomas M (2017). AURKA, DLGAP5, TPX2, KIF11 and CKAP5: Five specific mitosis-associated genes correlate with poor prognosis for non-small cell lung cancer patients. Int J Oncol.

[67] Li S, Xuan Y, Gao B, Sun X, Miao S, Lu T (2018). Identification of an eight-gene prognostic signature for lung adenocarcinoma. Cancer Manag Res.

[68] Zhou Z, Cheng Y, Jiang Y, Liu S, Zhang M, Liu J (2018). Ten hub genes associated with progression and prognosis of pancreatic carcinoma identified by co-expression analysis. Int J Biol Sci.

[69] Burks J, Fleury A, Livingston S, Smith JP (2019). ISG15 pathway knockdown reverses pancreatic cancer cell transformation and decreases murine pancreatic tumor growth via downregulation of PDL-1 expression. Cancer Immunol Immunother.

[70] Sun J, Yan J, Qiao HY, Zhao FY, Li C, Jiang JY (2020). Loss of TRIM29 suppresses cancer stem cell-like characteristics of PDACs via accelerating ISG15 degradation. Oncogene.

