# Supplementary material for: Identification of hub genes and their novel diagnostic and prognostic significance in pancreatic adenocarcinoma
Source: Cancer Biol Med. 2021 Aug 17;19(7):1029–46. doi: 10.20892/j.issn.2095-3941.2020.0516 (PMC9334760; doi:10.20892/j.issn.2095-3941.2020.0516)
Supplement: Supplementary file 1 [file cbm-19-1029-s001.pdf]

Supplementary materials

Supplementary materials and methods

Tissue microarray (TMA) and immunohistochemical analysis of disks large-associated protein 5 (DLGAP5)

The TMA contains well-documented follow-up and clinicopathological information, all of which were obtained with patient informed consent. The use of clinical samples was approved by the local Ethics Committee. TMA and ethical approval documentation were provided by Shanghai Outdo Biotech. The expression of DLGAP5 was analyzed using a TMA, which contained 51 cases of primary pancreatic ductal adenocarcinoma (PDAC) tissues and 36 cases of matched adjacent noncancerous tissues with 1 point for each tissue. Standard immunohistochemistry (IHC) procedures were performed on the TMA slide using DLGAP5 antibody (#sc-377004, 1:100; Santa Cruz Biotechnology). The staining intensity score was determined as no staining (score = 0), light yellow (score = 1), yellow (score = 2), and dark brown (score = 3), while the percentage score of positive cells was determined as < 5% (score = 0), 5%–25% (score = 1), 26%–50% (score = 2), 51%–75% (score = 3), and > 75% (score = 4)[1]. The IHC_{DLGAP5} score was determined by multiplying the intensity score and the percentage score. IHC_{DLGAP5} scores were classified as follows: negative (0); weakly positive (1 to 2); moderately positive (3 to 5); and strongly positive (6 to 12), and were independently evaluated by 2 pathologists blinded to the clinical information.

ELISA of serum IFN-stimulated gene 15 (ISG15)

Briefly, the standard was reconstituted with 1.0 mL of sample diluent. The reconstitution produced a stock solution of 50 ng/mL and diluted standard solutions of 25 ng/mL, 12.5 ng/mL, 6.25 ng/mL, 3.12 ng/mL, 1.56 ng/mL, and 0.78 ng/mL. The high standard had a concentration of 50 ng/mL. The zero standard had a concentration of 0 ng/mL. A total of 100 μL of standard and serum samples were added to 96-well plates and incubated in the dark at 37 °C for 2 h. After removing the liquid from each well, 100 μL of biotin-antibody (1×) was added to each well and then incubated in the dark at the same temperature for another 1 h. A total of 96 wells were dried and washed 3 times. After drying, 100 μL of horseradish peroxidase-avidin (1×) was added to each well and then incubated in the dark at the same temperature for another 1 h; then, each well was washed and dried 5 times. Then, 90 μL of 3,3′,5,5′-tetramethylbenzidine substrate was added to each well and incubated in the dark at the same temperature for another 15 min to 30 min. A total of 50 μL of stop solution was added to each well, and the absorbance of each well was then measured at 450 nm within 5 min using a microplate reader (BioTek, Winooski, VT, USA). We used known standards, including the high standard, the diluted standards, and the zero standard, to generate a calibration curve. The protein expression levels of ISG15 were calculated by comparing absorbance readings against the calibration curve. The detection range of serum ISG15 was 0.78 ng/mL to 50 ng/mL. Samples that yielded readings outside the detection upper limit were further diluted. The readings below the lower limit of detection were all less than or equal to 0.78 ng/mL. All samples and standards were tested in duplicate. All tests were blind to the disease status.

Table S1 GO and KEGG pathway enrichment analyses of 752 DEGs

Term	Description	Count in gene set	*P*-value	FDR
Biological process				
GO:0030198	Extracellular matrix organization	40	6.22e-17	2.00e-13
GO:0060337	Type I interferon signaling pathway	24	1.63e-16	2.00e-13
GO:0007155	Cell adhesion	60	2.15e-15	3.81e-12
GO:0030574	Collagen catabolic process	20	3.85e-12	6.96e-09
GO:0030199	Collagen fibril organization	15	1.52e-10	2.75e-07
GO:0009615	Response to virus	22	2.40e-09	4.33e-06
GO:0035987	Endodermal cell differentiation	11	4.74e-08	8.57e-05
GO:0051607	Defense response to virus	25	4.79e-08	8.65e-05
GO:0042060	Wound healing	17	9.73e-08	1.76e-04
GO:0045071	Negative regulation of viral genome replication	12	3.30e-07	5.96e-04
GO:0050900	Leucocyte migration	20	4.05e-07	7.32e-04
GO:0001525	Angiogenesis	26	3.81e-06	6.88e-03
Molecular function				
GO:0005178	Integrin binding	20	2.69e-08	4.22e-05
GO:0005518	Collagen binding	15	6.59e-08	1.04e-04
GO:0005201	Extracellular matrix structural constituent	14	1.87e-06	2.93e-03
GO:0043236	Laminin binding	9	3.47e-06	5.45e-03
Cell component				
GO:0070062	Extracellular exosome	215	1.38e-24	1.95e-21
GO:0005615	Extracellular space	127	3.99e-21	5.67e-18
GO:0031012	Extracellular matrix	48	1.13e-16	1.55e-13
GO:0005578	Proteinaceous extracellular matrix	41	2.21e-13	3.14e-10
GO:0005925	Focal adhesion	50	4.07e-13	5.78e-10
GO:0009986	Cell surface	58	7.66e-12	1.09e-08
GO:0005576	Extracellular region	113	6.28e-10	8.92e-07
GO:0005604	Basement membrane	16	3.13e-07	4.45e-04
GO:0005886	Plasma membrane	216	6.22e-07	8.84e-04
GO:0005856	Cytoskeleton	36	1.22e-06	1.74e-03
GO:0045121	Membrane raft	25	1.61e-06	2.28e-03
GO:0005788	Endoplasmic reticulum lumen	24	1.63e-06	2.32e-03
GO:0005887	Integral component of plasma membrane	90	3.59e-06	5.10e-03
KEGG pathway				
Hsa04512	ECM-receptor interaction	22	1.67e-10	2.16e-07
Hsa05146	Amoebiasis	21	4.35e-08	5.62e-05
Hsa04510	Focal adhesion	28	5.44e-07	7.02e-04
Hsa04145	Phagosome	22	3.60e-06	4.65e-03

GO, Gene Ontology; KEGG, Kyoto Encyclopedia of Genes and Genomes; DEGs, differentially expressed genes; FDR, false discovery rate; ECM, extracellular matrix.

Table S2 GO and KEGG pathway enrichment analyses of hub genes in the most densely connected module

Term	Description	Genes	*P*-value	FDR
Biological process				
GO:0060337	Type I interferon signaling pathway	*IFITM1, BST2, IFITM2, OAS3, HLA-A, RSAD2, OAS1, OAS2, HLA-B, STAT1, PSMB8, IFI35, ISG20, HLA-F, IRF9, IFIT3, IFIT1, IFI27, ISG15, XAF1, MX1, MX2, GBP2, IFI6*	2.07e-42	2.90e-39
GO:0051607	Defense response to virus	*IFITM1, BST2, IFITM2, OAS3, RSAD2, OAS1, IFI44L, OAS2, IFI16, STAT1, ISG20, IFIT3, IRF9, IFIT1, ISG15, DDX60, MX1, MX2, GBP1*	8.90e-23	1.25e-19
GO:0009615	Response to virus	*IFIT3, IFIT1, BST2, IFITM1, IFITM2, DDX60, OAS3, RSAD2, OAS1, IFI44, OAS2, MX1, MX2, ISG20*	4.78e-17	6.70e-14
GO:0045071	Negative regulation of viral genome replication	*IFIT1, ISG15, BST2, IFITM1, IFITM2, OAS3, RSAD2, OAS1, IFI16, MX1, ISG20*	8.28e-17	1.55e-13
GO:0060333	Interferon-γ-mediated signaling pathway	*IRF9, OAS3, HLA-A, OAS1, OAS2, HLA-B, STAT1, GBP2, GBP1, HLA-F*	2.31e-12	3.24e-09
GO:0035456	Response to interferon-β	*BST2, IFITM1, IFITM2, XAF1, STAT1*	1.59e-08	2.24e-05
GO:0000070	Mitotic sister chromatid segregation	*MAD2L1, NEK2, ZWINT, NUSAP1, NDC80, SMC4*	2.08e-08	2.92e-05
GO:0051301	Cell division	*CCNB1, FAM83D, CDK1, MAD2L1, NEK2, ZWINT, CKS2, CENPF, NDC80, PTTG1, CCNA2, SMC4*	2.62e-08	3.67e-05
GO:0007067	Mitotic nuclear division	*FAM83D, CDK1, NEK2, CENPF, NDC80, ANLN, PTTG1, CEP55, CCNA2, ASPM*	1.64e-07	2.30e-04
GO:0000281	Mitotic cytokinesis	*CKAP2, NUSAP1, ANLN, CEP55, RACGAP1*	2.85e-06	4.01e-03
GO:0035455	Response to interferon-α	*BST2, IFITM1, IFITM2, MX2*	4.61e-06	6.48e-03
Molecular function				
GO:0005515	Protein binding	*PRC1, IFITM1, NEK2, OAS3, RSAD2, OAS1, CEP55, OAS2, PTTG1, IFI35, FAM83D, ISG15, DDX60, MX1, TOP2A, CCNA2, MX2, CDK1, BST2, DTL, DLGAP5, GMNN, HLA-A, CENPF, NUSAP1, UBE2L6, NDC80, IFI16, RACGAP1, CENPK, STAT1, ECT2, PSMB8, SMC4, IFIT3, CCNB1, IRF9, IFIT1, MAD2L1, RRM2, ZWINT, CKS2, CENPU, KPNA2, GBP2, IFI6, MELK, GBP1*	1.85e-07	2.11e-04
Cell component				
GO:0005829	Cytosol	*PRC1, NEK2, OAS3, OAS1, OAS2, PTTG1, IFI35, ISG15, XAF1, MX1, MX2, CDK1, GMNN, CENPF, UBE2L6, NDC80, IFI16, RACGAP1, CENPK, STAT1, ECT2, PSMB8, SMC4, IRF9, CCNB1, IFIT3, IFIT1, MAD2L1, RRM2, ZWINT, CENPU, KPNA2, GBP2, GBP1*	4.72e-11	5.33e-08
GO:0005737	Cytoplasm	*PRC1, NEK2, OAS3, IFI44L, OAS1, OAS2, PTTG1, ISG20, FAM83D, DDX60, MX1, TOP2A, CCNA2, MX2, ASPM, CKAP2, CDK1, BST2, DTL, DLGAP5, GMNN, CENPF, NUSAP1, IFI44, IFI16, RACGAP1, STAT1, ECT2, PSMB8, SMC4, IRF9, CCNB1, IFIT3, IFIT1, RRM2, ZWINT, KPNA2*	1.19e-07	1.35e-04
GO:0030496	Midbody	*CDK1, PRC1, NEK2, CENPF, CEP55, RACGAP1, ECT2, ASPM*	1.68e-07	1.90e-04
GO:0005634	Nucleus	*PRC1, NEK2, OAS1, OAS2, PTTG1, IFI35, ISG20, XAF1, MX1, TOP2A, CCNA2, MX2, ASPM, CDK1, DTL, DLGAP5, GMNN, CENPF, NUSAP1, NDC80, IFI16, RACGAP1, CENPK, STAT1, ECT2, PSMB8, SMC4, IRF9, CCNB1, MAD2L1, RRM2, ZWINT, CENPU, KPNA2, MELK*	4.39e-06	5.00e-03

Table S2 Continued

Term	Description	Genes	*P*-value	FDR
GO:0005654	Nucleoplasm	*CDK1*, *PRC1*, *DTL*, *GMNN*, *OAS3*, *UBE2L6*, *CENPF*, *IFI44L*, *ANLN*, *IFI16*, *RACGAP1*, *CENPK*, *STAT1*, *PSMB8*, *SMC4*, *ISG20*, *IRF9*, *CCNB1*, *ISG15*, *RRM2*, *CENPU*, *TOP2A*, *CCNA2*, *KPNA2*	5.87e-06	6.63e-03
GO:0000777	Condensed chromosome kinetochore	*MAD2L1*, *NEK2*, *ZWINT*, *NDC80*, *CENPU*, *CENPK*	8.29e-06	9.37e-03
KEGG pathway				
Hsa05168	Herpes simplex infection	*IRF9*, *CDK1*, *IFIT1*, *OAS3*, *HLA-A*, *OAS1*, *OAS2*, *HLA-B*, *STAT1*, *HLA-F*	1.22e-09	1.15e-06
Hsa05164	Influenza A	*IRF9*, *OAS3*, *RSAD2*, *OAS1*, *OAS2*, *MX1*, *STAT1*, *KPNA2*	5.06e-07	4.76e-04

GO, Gene Ontology; KEGG, Kyoto Encyclopedia of Genes and Genomes; FDR, false discovery rate.

Table S3 Some hub genes were related to immune cell infiltration

Genes	Tumor purity		B cells		CD8+ T cells		CD4+ T cells		Macrophages		Neutrophils		Dendritic cells	
	P-value	Correlation	*P*-value	Partial correlation	*P*-value	Partial correlation	*P*-value	Partial correlation	*P*-value	Partial correlation	*P*-value	Partial correlation	*P*-value	Partial correlation
ISG15	0.064	−0.142	0.035	−0.072	0.069	−0.140	4.0e-05	0.311	0.688	−0.031	4.0e-04	0.270	0.020	0.178
IFI6	0.036	−0.160	0.235	−0.091	0.223	−0.094	0.002	0.240	0.694	−0.030	0.002	0.235	0.173	0.105
RSAD2	3.0e-04	−0.271	0.036	0.161	9.0e-05	0.296	0.005	0.214	2.0e-08	0.415	2.9e-15	0.556	6.0e-14	0.534
NUSAP1	0.688	−0.031	0.003	0.228	0.022	0.176	0.003	−0.227	0.812	−0.018	0.077	0.136	0.004	0.218
DTL	0.928	0.007	0.025	0.171	0.017	0.182	0.002	−0.235	0.667	0.033	0.339	0.074	0.006	0.211
CENPK	0.547	0.046	0.053	0.148	0.063	0.142	0.027	−0.170	0.745	−0.025	0.085	0.132	0.012	0.191
OAS1	0.110	−0.122	0.414	0.063	0.116	0.121	0.675	0.032	0.537	0.048	5.9e-05	0.302	0.015	0.185
CDK1	0.801	−0.019	0.304	0.079	0.385	0.067	0.001	−0.245	0.266	−0.086	0.472	0.055	0.116	0.121
CCNA2	0.552	0.046	0.009	0.199	0.104	0.125	9.0e-04	−0.254	0.662	−0.034	0.606	0.040	0.006	0.211
CENPF	0.728	0.027	0.103	0.125	0.052	0.149	2.0e-04	−0.282	0.683	0.031	0.442	0.059	0.045	0.154
TOP2A	0.954	0.004	0.008	0.202	0.002	0.231	1.0e-04	−0.294	0.182	0.103	0.118	0.120	0.001	0.250
DLGAP5	0.920	−0.008	0.022	0.175	0.052	0.149	0.005	−0.215	0.928	−0.007	0.199	0.099	0.003	0.230
PRC1	0.953	−0.005	0.046	0.153	0.055	0.147	7.0e-04	−0.258	0.972	0.003	0.423	0.062	0.021	0.177
ASPM	0.685	0.031	0.031	0.165	0.043	0.155	0.001	−0.246	0.989	0.001	0.310	0.078	0.024	0.173
NDC80	0.966	0.003	0.012	0.193	0.042	0.156	0.116	−0.121	0.130	0.116	0.019	0.179	0.001	0.244
CEP55	0.844	−0.015	0.276	0.084	0.220	0.094	9.0e-04	−0.253	0.397	−0.065	0.401	0.065	0.037	0.160
DDX60	0.054	−0.147	0.002	0.234	3.0e-06	0.350	0.865	0.013	0.002	0.239	6.6e-10	0.450	9.0e-08	0.396
RACGAP1	0.801	−0.019	0.006	0.210	3.0e-04	0.276	0.002	−0.234	0.013	0.191	0.052	0.149	5.0e-04	0.265
ECT2	0.597	−0.041	0.017	0.183	0.023	0.174	3.0e-04	−0.272	0.732	0.026	0.106	0.124	0.009	0.199
CKS2	0.329	0.075	0.428	0.061	0.716	0.028	0.003	−0.227	0.128	−0.117	0.821	0.017	0.198	0.099
ANLN	0.904	0.009	0.097	0.127	0.014	0.187	3.0e-04	−0.275	0.333	0.074	0.474	0.055	0.008	0.203
NEK2	0.646	0.035	0.178	0.103	0.496	0.052	1.0e-04	−0.295	0.219	−0.095	0.781	−0.021	0.326	0.076
BST2	0.009	−0.198	0.063	0.143	0.090	0.130	0.001	0.252	0.076	0.136	1.3e-07	0.390	2.0e-06	0.352
CCNB1	0.922	−0.007	0.089	0.130	0.259	0.087	0.015	−0.187	0.508	−0.051	0.431	0.061	0.046	0.153
CENPU	0.875	−0.012	0.030	0.167	0.325	0.076	0.022	−0.176	0.249	−0.089	0.584	0.042	0.050	0.150

Table S3 Continued

Genes	Tumor purity		B cells		CD8+ T cells		CD4+ T cells		Macrophages		Neutrophils		Dendritic cells	
	P-value	Correlation	*P*-value	Partial correlation	*P*-value	Partial correlation	*P*-value	Partial correlation	*P*-value	Partial correlation	*P*-value	Partial correlation	*P*-value	Partial correlation
CKAP2	0.980	0.002	2.0e-04	0.279	3.0e-07	0.381	0.028	−0.169	8.0e-05	0.297	0.020	0.178	2.0e-07	0.384
FAM83D	0.825	−0.017	0.069	0.139	0.001	0.248	0.019	−0.181	0.023	0.174	0.059	0.145	0.003	0.228
GBP1	7.0e-05	−0.298	2.0e-07	0.386	1.0e-12	0.509	0.003	0.232	2.0e-15	0.558	1.1e-20	0.635	4.0e-27	0.706
GBP2	0.062	−0.143	4.0e-05	0.310	9.0e-09	0.422	0.271	0.085	3.0e-04	0.275	1.5e-07	0.388	6.0e-11	0.473
GMNN	0.127	−0.117	0.345	0.073	0.205	0.097	0.260	−0.087	0.452	−0.058	0.813	0.018	0.573	0.043
HLA-A	0.202	−0.098	0.006	0.210	0.462	−0.057	7.0e-05	0.301	0.764	0.023	7.0e-04	0.257	5.0e-04	0.265
HLA-B	0.084	−0.132	2.0e-05	0.317	0.020	0.178	2.0e-05	0.322	2.0e-04	0.285	9.9e-13	0.510	1.0e-13	0.528
HLA-F	0.179	−0.103	2.0e-06	0.354	0.851	0.014	3.0e-06	0.348	0.934	−0.006	2.9e-06	0.349	8.0e-06	0.335
IFI16	0.052	−0.148	8.0e-07	0.366	4.0e-10	0.456	0.005	0.213	4.0e-17	0.586	4.3e-19	0.614	5.0e-20	0.627
IFI27	0.030	−0.166	0.349	0.072	0.947	−0.005	0.184	0.103	0.546	−0.046	0.003	0.228	0.011	0.195
IFI35	0.005	−0.214	0.220	0.094	0.611	0.039	0.002	0.235	0.878	0.012	2.9e-05	0.314	0.001	0.242
IFI44	0.008	−0.201	0.213	0.096	0.012	0.191	4.0e-05	0.311	0.004	0.222	2.4e-11	0.482	6.0e-06	0.338
IFI44L	0.002	−0.232	0.069	0.139	0.001	0.251	7.0e-04	0.257	0.001	0.245	2.3e-11	0.483	1.0e-06	0.364
IFIT1	0.001	−0.242	0.683	0.031	0.012	0.191	0.002	0.237	0.003	0.229	1.5e-08	0.417	3.0e-05	0.315
IFIT3	2.0e-04	−0.280	0.085	0.132	1.0e-04	0.288	0.010	0.199	2.0e-04	0.283	2.0e-13	0.524	4.0e-10	0.454
IFITM1	0.031	−0.165	0.025	0.171	0.087	0.131	5.0e-05	0.307	0.007	0.204	5.2e-11	0.475	1.0e-08	0.421
IFITM2	0.126	−0.117	0.219	0.095	0.872	−0.012	6.0e-06	0.341	0.098	0.127	5.0e-04	0.264	0.023	0.174
IRF9	0.012	−0.191	0.031	0.165	0.005	0.213	0.020	0.179	0.034	0.162	3.1e-07	0.379	5.0e-06	0.342
ISG20	0.681	−0.032	0.020	0.177	0.129	−0.116	0.005	0.217	0.258	−0.087	0.022	0.175	0.026	0.171
KPNA2	0.392	−0.066	8.0e-04	0.254	0.001	0.245	0.641	−0.036	7.0e-04	0.255	0.004	0.219	1.0e-06	0.361
MAD2L1	0.554	0.045	0.011	0.194	0.040	0.157	3.0e-05	−0.316	0.996	< 0.001	0.658	0.034	0.002	0.234
MELK	0.792	0.020	0.090	0.130	0.654	0.034	0.051	−0.150	0.693	−0.030	0.724	0.027	0.094	0.129
MX1	0.004	−0.218	0.078	0.135	0.140	0.113	2.0e-04	0.287	0.076	0.136	2.0e-08	0.413	9.0e-06	0.333
MX2	0.070	−0.138	0.014	0.187	0.118	0.120	0.001	0.252	0.033	0.163	4.4e-08	0.404	6.0e-06	0.340
OAS2	0.004	−0.219	0.007	0.204	0.005	0.214	0.007	0.208	0.001	0.245	3.7e-13	0.519	1.0e-08	0.418

Table S3 Continued

Genes	Tumor purity		B cells		CD8+ T cells		CD4+ T cells		Macrophages		Neutrophils		Dendritic cells	
	P-value	Correlation	*P*-value	Partial correlation	*P*-value	Partial correlation	*P*-value	Partial correlation	*P*-value	Partial correlation	*P*-value	Partial correlation	*P*-value	Partial correlation
OAS3	0.017	−0.182	0.036	0.160	3.0e-04	0.273	0.196	0.100	0.001	0.247	5.3e-11	0.475	7.0e-08	0.399
PSMB8	0.113	−0.121	6.0e-07	0.372	0.516	0.050	0.052	0.150	0.534	−0.048	0.004	0.218	3.0e-05	0.311
PTTG1	0.335	0.074	0.593	0.041	0.266	−0.086	0.076	−0.137	0.010	−0.197	0.860	0.014	0.262	0.086
RRM2	0.989	−0.001	0.031	0.165	0.176	0.104	0.022	−0.176	0.677	−0.032	0.402	0.065	0.004	0.220
SMC4	0.675	−0.032	5.0e-05	0.305	6.0e-09	0.427	0.026	−0.172	7.0e-05	0.300	9.3e-05	0.294	1.0e-08	0.418
STAT1	0.004	−0.221	3.0e-04	0.275	3.0e-12	0.501	0.084	0.133	3.0e-10	0.458	1.6e-16	0.577	9.0e-19	0.609
UBE2L6	4.0e-04	−0.266	7.0e-05	0.300	9.0e-11	0.470	0.011	0.195	2.0e-07	0.388	1.2e-17	0.594	8.0e-19	0.610
XAF1	0.071	−0.138	0.050	0.150	0.086	0.132	5.0e-05	0.307	0.004	0.218	2.6e-10	0.459	3.0e-05	0.311
ZWINT	0.868	−0.013	0.134	0.115	0.997	< 0.001	0.043	−0.156	0.055	−0.147	0.699	0.030	0.160	0.108

CKAP2, *GBP1*, *IFI16*, *IRF9*, *OAS2*, *RSAD2*, *SMC4* and *UBE2L6* were significantly correlated with the infiltration levels of all 6 immune cells (B cells, CD4+ T cells, CD8+ T cells, macrophages, neutrophils, and dendritic cells) (P-value < 0.05).

Table S4 Differential mRNA expression of *HLA-A*, *IFI27*, *ISG15*, and *KPNA2* in PA *vs*. normal pancreatic tissues from the Oncomine database

Genes	PA, *n*	Normal, *n*	Fold change	*t*-test, T	*P*-value	Gene rank	Reference
HLA-A	11	6	2.587	6.086	1.10e-05*	82 (top 1%)	[2]
	12	5	4.754	6.566	3.01e-05*	137 (top 1%)	[3]
	39	39	2.362	6.178	7.49e-08*	1,937 (top 10%)	[4]
	10	5	6.203	9.179	1.94e-04*	270 (top 6%)	[5]
	36	16	1.852	3.854	5.46e-04*	2,453 (top 13%)	[6]
	8	6	1.342	1.352	0.104	3,550 (top 23%)	[7]
	11	11	1.979	1.029	0.162	5,641 (top 32%)	[8]
	24	25	−1.163	−1.835	0.964	17,218 (top 97%)	[9]
IFI27	12	5	5.076	6.292	7.87e-06*	81 (top 1%)	[3]
	36	16	10.237	8.083	1.24e-09*	103 (top 1%)	[6]
	39	39	4.912	7.547	9.74e-11*	584 (top 3%)	[4]
	11	11	8.843	2.685	0.009*	403 (top 3%)	[8]
	10	5	765.104	4.045	0.008*	778 (top 15%)	[5]
	11	6	4.937	3.780	0.001*	1,004 (top 8%)	[2]
	8	6	1.311	0.626	0.274	5,727 (top 37%)	[7]
	24	25	1.013	0.063	0.475	10,397 (top 59%)	[9]
ISG15	12	5	4.646	7.856	5.48e-07*	19 (top 1%)	[3]
	39	39	3.726	8.032	1.21e-11*	397 (top 3%)	[4]
	36	16	4.965	6.164	3.10e-07*	413 (top 3%)	[6]
	11	11	4.546	2.458	0.012*	539 (top 4%)	[8]
	10	5	2.462	3.394	0.003*	562 (top 11%)	[5]
	11	6	3.717	3.020	0.006*	1,827 (top 15%)	[2]
	24	25	1.110	0.393	0.348	8,233 (top 47%)	[9]
	8	6	−1.367	−2.451	0.985	14,198 (top 91%)	[7]
KPNA2	11	6	4.194	6.322	7.03e-06*	65 (top 1%)	[2]
	36	16	2.485	4.740	7.30e-05*	1,497 (top 8%)	[6]
	39	39	2.644	6.451	2.16e-08*	1,587 (top 9%)	[4]
	10	5	2.816	3.186	0.014*	933 (top 18%)	[5]
	24	25	1.428	1.911	0.031*	1,122 (top 7%)	[9]
	12	5	2.588	2.914	0.013*	1,832 (top 13%)	[3]
	11	11	2.042	1.465	0.084	3,220 (top 19%)	[8]
	8	6	−1.449	−3.619	0.998	15,091 (top 96%)	[7]

Differential mRNA expression of *HLA-A* with a **P*-value < 0.05 was found in 5 datasets. Differential mRNA expression of *IFI27* with a **P*-value < 0.05 was found in 6 datasets. Differential mRNA expression of *ISG15* with a **P*-value < 0.05 was found in 6 datasets. Differential mRNA expression of *KPNA2* with a **P*-value < 0.05 was found in 6 datasets. PA, pancreatic adenocarcinoma; *n*, number; Ref., references.

Table S5 Relationship between ISG15 protein levels and the clinical characteristics of PA patients from the TMA

Clinicopathologic variable	ISG15		*P*-value
	Low, *n* (%)	High, *n* (%)	
Age (y)			0.738
≥ 63	9 (50.0)	9 (50.0)	
< 63	10 (58.8)	7 (41.2)	
Gender			0.727
Male	11 (50.0)	11 (50.0)	
Female	8 (61.5)	5 (38.5)	
Grade			1.000
I–II	12 (52.2)	11 (47.8)	
III	7 (58.3)	5 (41.7)	
T stage			0.139
T2	4 (40.0)	6 (60.0)	
T3	14 (70.0)	6 (30.0)	
N stage			0.724
N0	13 (59.1)	9 (40.9)	
N1	6 (50.0)	6 (50.0)	
M stage			0.209
M0	12 (60.0)	8 (40.0)	
M1	2 (28.6)	5 (71.4)	

Pathological diagnosis was confirmed according to the Staging Manual of the American Joint Committee on Cancer staging system, 8th edition. PA, pancreatic adenocarcinoma; TMA, tissue microarray; *n*, number; y, years; T, tumor; N, node; M, metastasis.

Table S6 Relationship between DLGAP5 expression and the clinical characteristics of PA patients from the TMA

Clinicopathologic variable	DLGAP5		*P*-value
	Negative, *n* (%)	Weakly positive, *n* (%)	
Age (y)			0.716
≥ 66	17 (58.6)	12 (41.4)	
< 66	14 (63.6)	8 (36.4)	
Gender			0.244
Male	15 (53.6)	13 (46.4)	
Female	16 (69.6)	7 (30.4)	
Grade			0.008*
I	5 (100.0)	0 (0.0)	
II	25 (64.1)	14 (35.9)	
III	1 (14.3)	6 (85.7)	
T stage			0.415
T1	2 (100.0)	0 (0.0)	
T2	10 (55.6)	8 (44.4)	
T3	18 (66.7)	9 (33.3)	
N stage			0.847
N0	17 (60.7)	11 (39.3)	
N1	11 (57.9)	8 (42.1)	
M stage			0.279
M0	28 (59.6)	19 (40.4)	
M1	3 (100.0)	0 (0.0)	

Pathological diagnosis was confirmed according to the Staging Manual of the American Joint Committee on Cancer staging system, 8th edition. PA, pancreatic adenocarcinoma; TMA, tissue microarray; *n*, number; y, years; T, tumor; N, node; M, metastasis; **P*-value < 0.05.

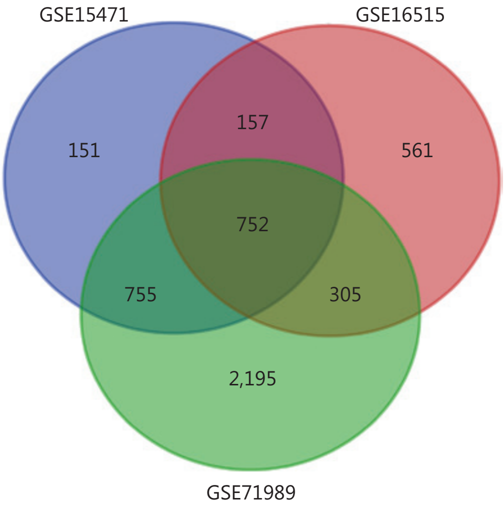

Figure S1 Venn diagram showing 752 overlapping DEGs from 3 datasets of GEO. DEGs were screened with a |log FC| > 1 and adjusted *P*-value < 0.01 among the datasets of the mRNA expression profiles (GSE15471, GSE16515 and GSE71989). DEGs, differentially expressed genes; GEO, Gene Expression Omnibus; FC, fold change.

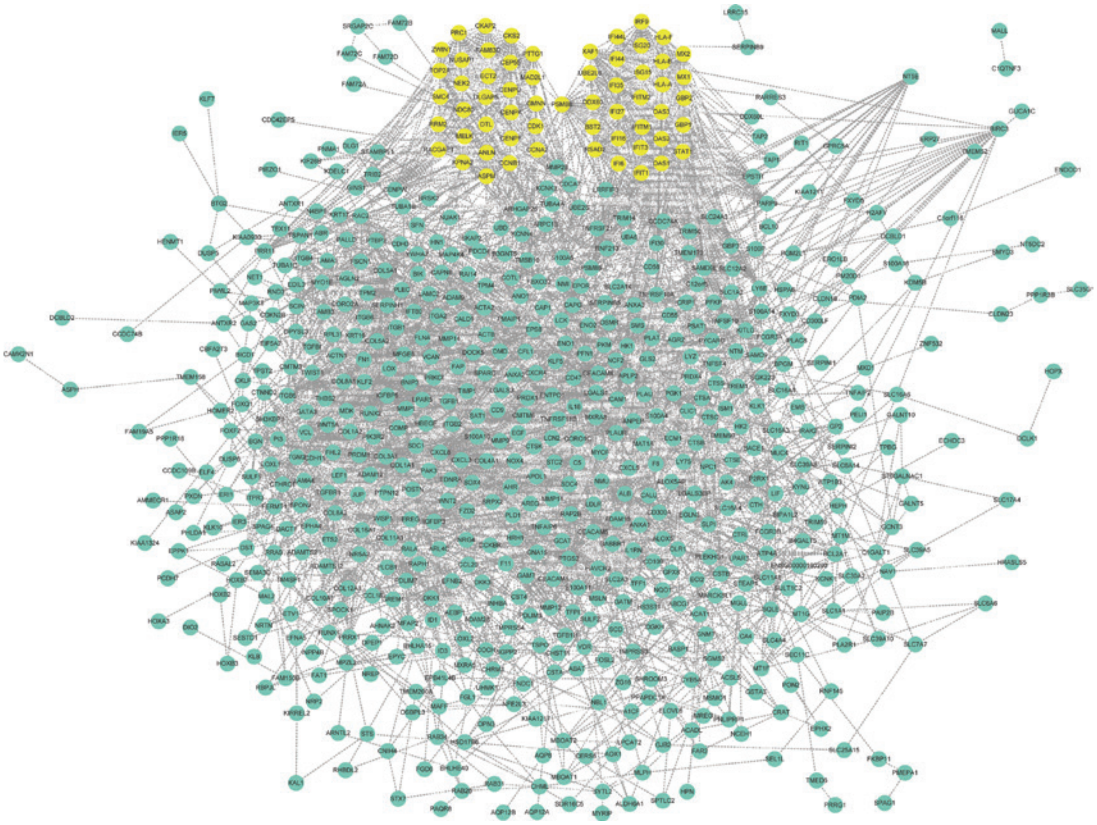

Figure S2 The PPI network of 752 DEGs. The network was visualized using Cytoscape. The yellow module is the most densely connected module from this PPI network. The yellow module was magnified as shown in **Figure 3**. DEGs, differentially expressed genes; PPI, protein-protein interaction.

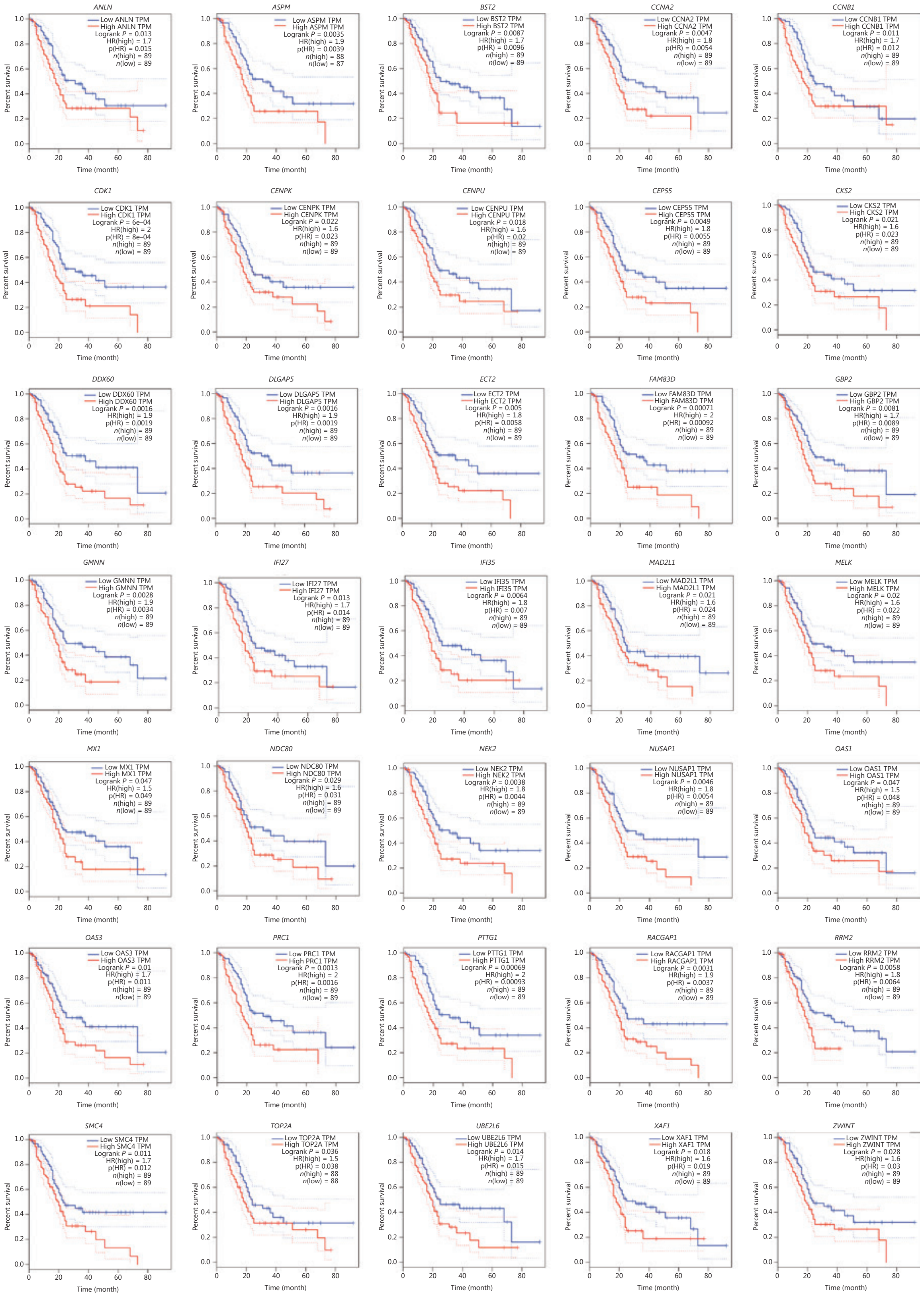

Figure S3 Correlation of high expression levels of individual hub genes with poor OS of PA patients. Kaplan-Meier survival curves were generated using the online GEPIA platform. The OS curve for patients with high expression levels of hub genes is indicated by a red solid line.

The OS curve for patients with low expression levels of hub genes is indicated by a blue solid line. The 95% confidence interval is shown by a dashed line. $P < 0.05$ in the log-rank test was considered statistically significant. OS, overall survival; PA, pancreatic adenocarcinoma; GEPIA, Gene Expression Profiling Interactive Analysis.

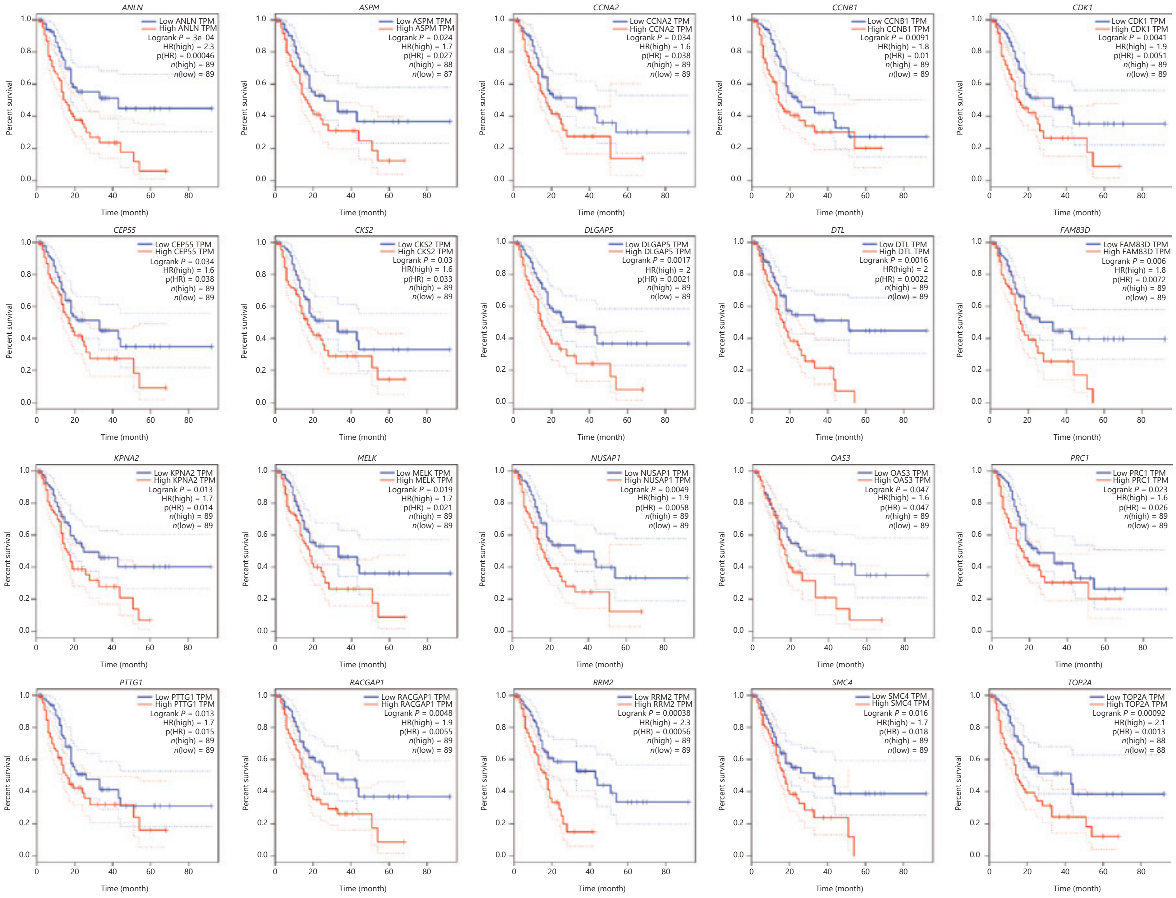

Figure S4 Correlation of high expression levels of individual hub genes with poor DFS of PA patients. Kaplan-Meier survival curves were generated using the online GEPIA platform. The DFS curve for patients with high expression levels of hub genes is indicated by a red solid line. The DFS curve for patients with low expression levels of hub genes is indicated by a blue solid line. The 95% confidence interval is shown by a dashed line. $P < 0.05$ in the log-rank test was considered statistically significant. DFS, disease-free survival; PA, pancreatic adenocarcinoma; GEPIA, Gene Expression Profiling Interactive Analysis.

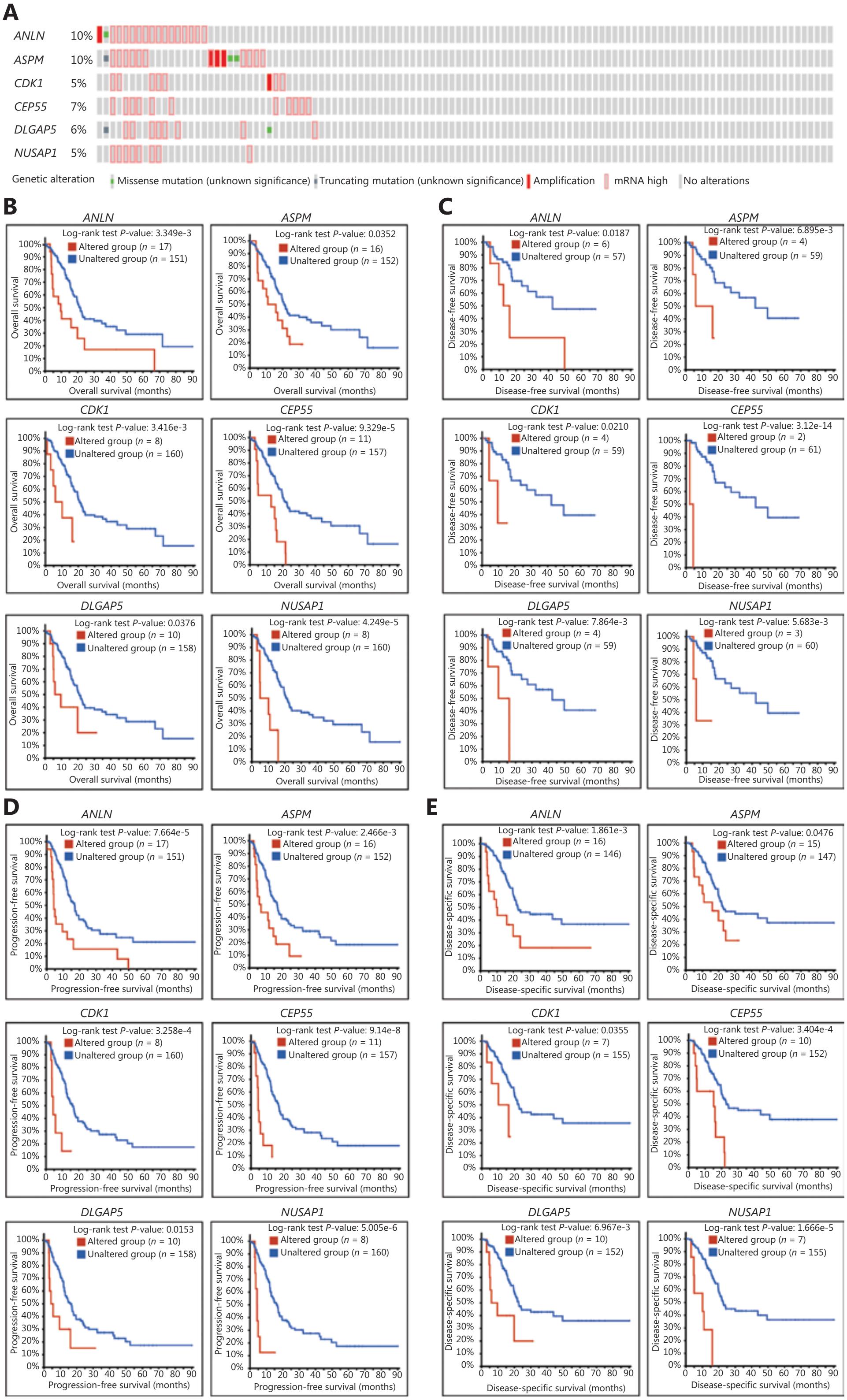

Figure S5 Survival analyses of hub genetic alterations performed using cBioPortal. (A) Hub genetic alterations in PA. *ANLN* was altered in 17 (10%) of 168 PA patients/samples. The alterations in *ANLN* consisted of amplifications, missense mutations, and high mRNA expression levels. *ASPM* was altered in 16 (10%) of 168 PA patients/samples. The alterations in *ASPM* consisted of amplifications, truncating mutations,

missense mutations, and high mRNA expression levels. *CDK1* was altered in 8 (5%) of 168 PA patients/samples. The alterations in *CDK1* consisted of amplification and high mRNA expression levels. *CEP55* was altered in 11 (7%) of 168 PA patients/samples. The alterations in *CEP55* consisted of high mRNA expression levels. *DLGAP5* was altered in 10 (6%) of 168 PA patients/samples. The alterations in *DLGAP5* consisted of truncating mutations, missense mutations and high mRNA expression levels. *NUSAP1* was altered in 8 (5%) of 168 PA patients/samples. The alterations in *NUSAP1* were high mRNA expression levels. (B) Altered individual hub genes were related to worse OS of PA patients. $P < 0.05$ in the log-rank test was considered statistically significant. Overall indicates overall patient survival status. (C) Altered individual hub genes were related to worse DFS of PA patients. $P < 0.05$ in the log-rank test was considered statistically significant. Disease-free survival indicates the DFS of patients since initial treatment. (D) Altered individual hub genes were related to worse PFS of PA patients. $P < 0.05$ in the log-rank test was considered statistically significant. Progression-free survival indicates the PFS of patients. (E) Altered individual hub genes were related to worse DSS of PA patients. $P < 0.05$ in the log-rank test was considered statistically significant. The time period of DSS usually starts at the time of PA diagnosis or at the beginning of treatment and ends at the time of death. PA, pancreatic adenocarcinoma; *n*, number; OS, overall survival; DFS, disease-free survival; PFS, progression-free survival; DSS, disease-specific survival.

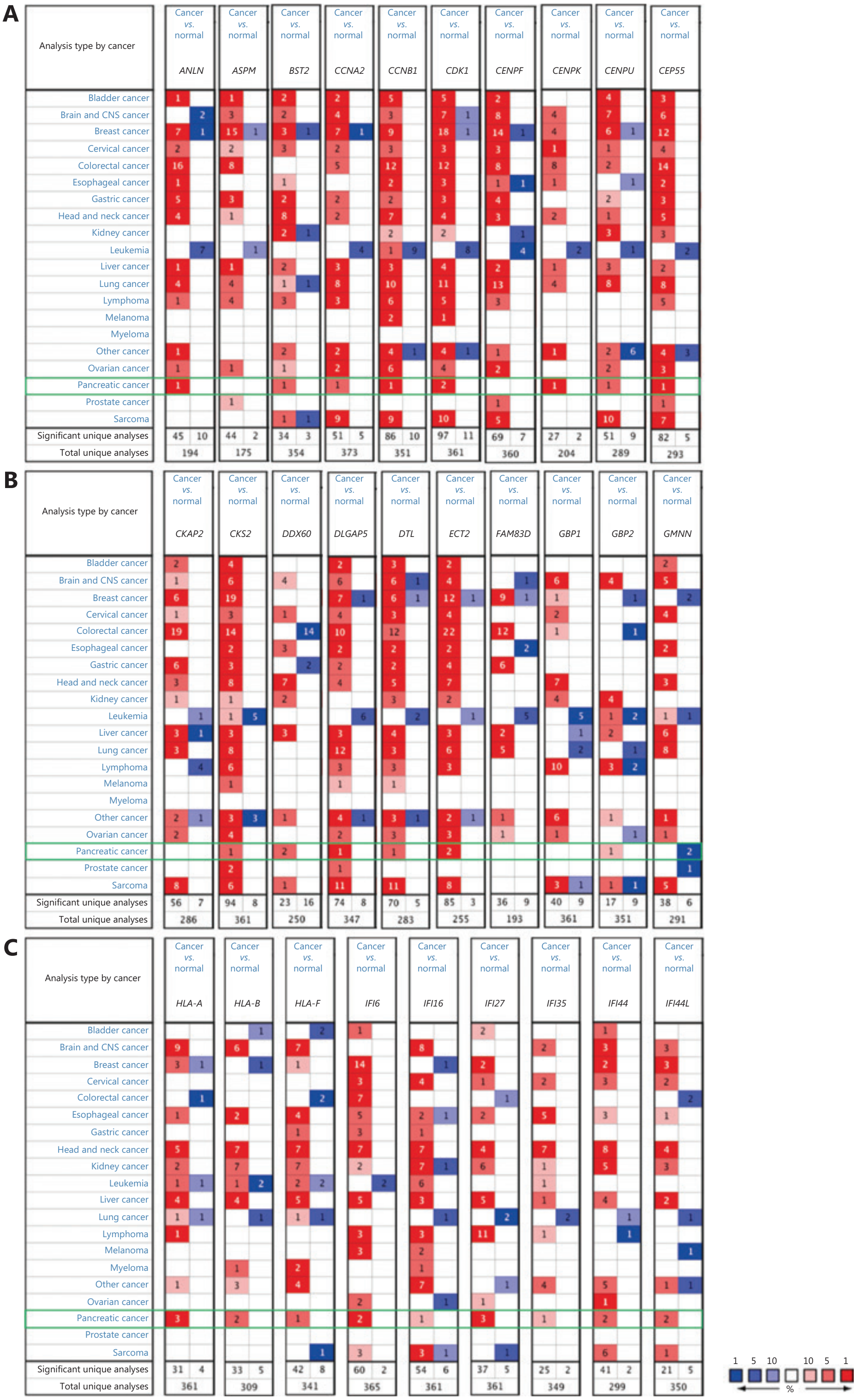

Figure S6 Continued

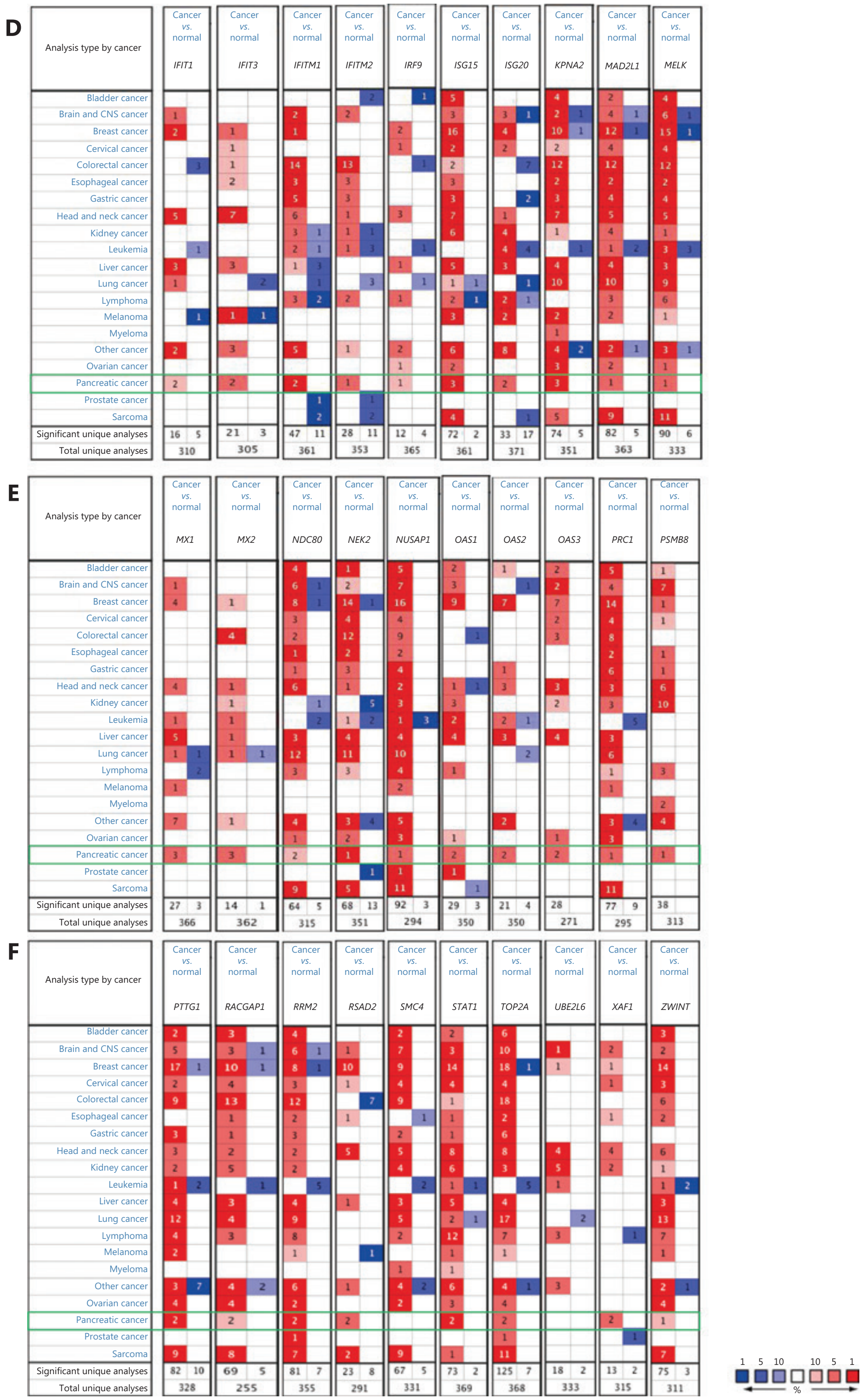

Figure S6 Heat maps of differential mRNA expression of individual hub genes in cancer *vs*. normal tissues from the Oncomine database. The thresholds ("*P*-value of 1E-4", "2-fold change", and "top 10% genes in gene rank") were used for screening up- and down-regulated expression of hub genes, which are shown in red and blue cells, respectively. The number in each cell represents the number of datasets that meet the filter criteria. The differential expression levels of the hub genes in PA are shown in the green frame. Color depth in the cell indicates the best gene rank percentile between the top 1% and top 10%. The differential expression levels of *ANLN*, *ASPM*, *BST2*, *CCNA2*, *CCNB1*, *CDK1*, *CENPF*,

CENPK, *CENPU*, and *CEP55* with different depth of color in the cell are shown in (A). The differential expression levels of *CKAP2*, *CKS2*, *DDX60*, *DLGAP5*, *DTL*, *ECT2*, *FAM83D*, *GBP1*, *GBP2*, and *GMNN* with different depth of color in the cell are shown in (B). The differential expression levels of *HLA-A*, *HLA-B*, *HLA-F*, *IFI6*, *IFI16*, *IFI27*, *IFI35*, *IFI44*, and *IFI44L* with different depth of color in the cell are shown in (C). The differential expression levels of *IFIT1*, *IFIT3*, *IFITM1*, *IFITM2*, *IRF9*, *ISG15*, *ISG20*, *KPNA2*, *MAD2L1*, and *MELK* with different depth of color in the cell are shown in (D). The differential expression levels of *MX1*, *MX2*, *NDC80*, *NEK2*, *NUSAP1*, *OAS1*, *OAS2*, *OAS3*, *PRC1*, and *PSMB8* with different depth of color in the cell are shown in (E). The differential expression levels of *PTTG1*, *RACGAP1*, *RRM2*, *RSAD2*, *SMC4*, *STAT1*, *TOP2A*, *UBE2L6*, *XAF1*, and *ZWINT* with different depth of color in the cell are shown in (F). PA, pancreatic adenocarcinoma.

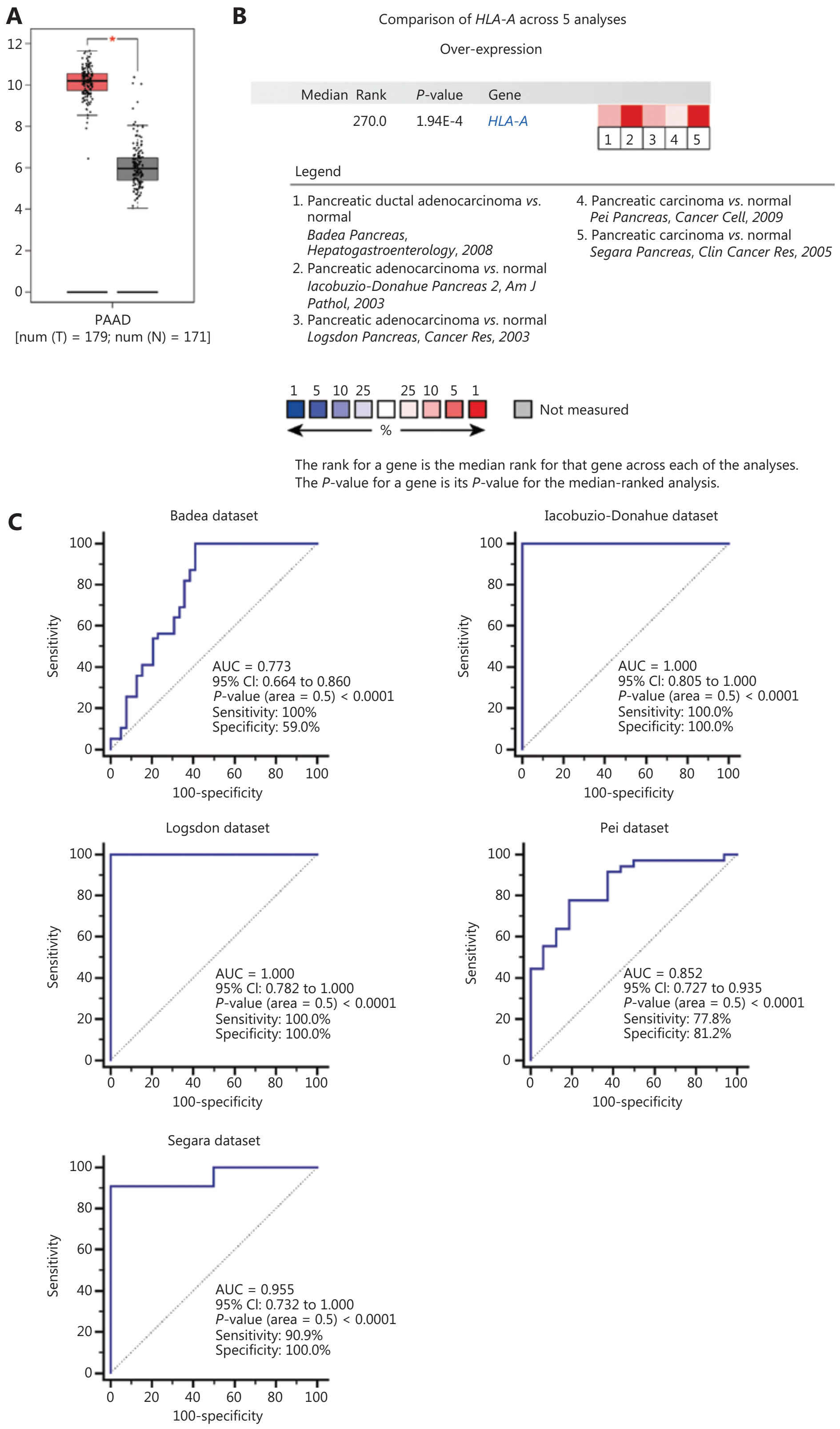

Figure S7 Analyses of *HLA-A* mRNA expression in PA *vs.* normal pancreatic tissues for diagnosis. (A) Analysis of *HLA-A* mRNA expression in PA *vs.* normal pancreatic tissues from TCGA and GTEx datasets. The following settings were used for the analysis: "expression on box plots", "gene = *HLA-A*", "|$\log_2$(FC)| cut-off = 1", "*P*-value cut-off = 0.01", "datasets = PAAD", "log scale = $\log_2$(TPM + 1)", "jitter size = 0.4", and "matched

TCGA normal and GTEx data". (B) Heat map of *HLA-A* expression in PA *vs*. normal tissues with a *P*-value < 0.05 from 5 independent datasets from Oncomine. The 5 datasets include the Badea dataset[4], Iacobuzio-Donahue dataset[3], Logsdon dataset[5], Pei dataset[6], and Segara dataset[2]. (C) ROC curve analysis of *HLA-A* expression with a *P*-value < 0.05 was used to diagnose PA from the 5 independent datasets from Oncomine. PA, pancreatic adenocarcinoma; T, tumor; N, normal; **P*-value < 0.01; TCGA, The Cancer Genome Atlas; GTEx, Genotype-Tissue Expression; FC, fold change; PAAD, pancreatic adenocarcinoma; TPM, transcripts per million; ROC, receiver operating characteristic; AUC, area under the ROC curve; CI, confidence interval.

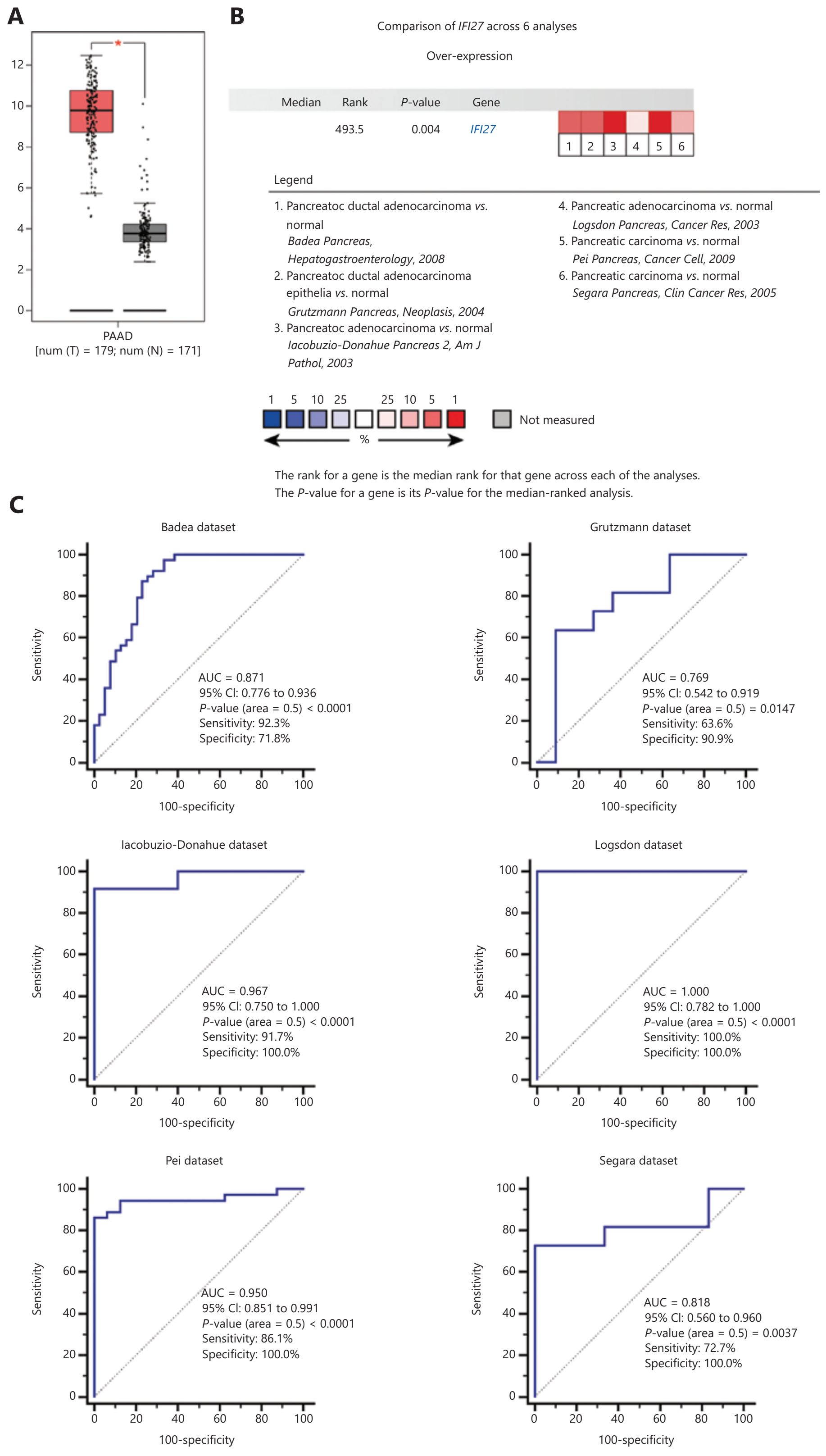

Figure S8 Analyses of *IFI27* mRNA expression in PA *vs.* normal pancreatic tissues for diagnosis. (A) Analysis of *IFI27* mRNA expression in PA *vs.* normal pancreatic tissues from TCGA and GTEx datasets. The following settings were used for the analysis: "expression on box plots", "gene = *IFI27*", "$|\log_2(FC)|$ cut-off = 1", "*P*-value cut-off = 0.01", "datasets = PAAD", "log scale = $\log_2(TPM + 1)$", "jitter size = 0.4", and "matched TCGA

normal and GTEx data". (B) Heat map of *IFI27* expression in PA *vs*. normal tissues with a *P*-value < 0.05 from 6 independent datasets from Oncomine. The 6 datasets include the Badea dataset[4], Grutzmann dataset[8], Iacobuzio-Donahue dataset[3], Logsdon dataset[5], Pei dataset[6], and Segara dataset[2]. (C) ROC curve analysis of *IFI27* expression with a *P*-value < 0.05 was used to diagnose PA from the 6 independent datasets from Oncomine. PA, pancreatic adenocarcinoma; T, tumor; N, normal; **P*-value < 0.01; TCGA, The Cancer Genome Atlas; GTEx, Genotype-Tissue Expression; FC, fold change; PAAD, pancreatic adenocarcinoma; TPM, transcripts per million; ROC, receiver operating characteristic; AUC, area under the ROC curve; CI, confidence interval.

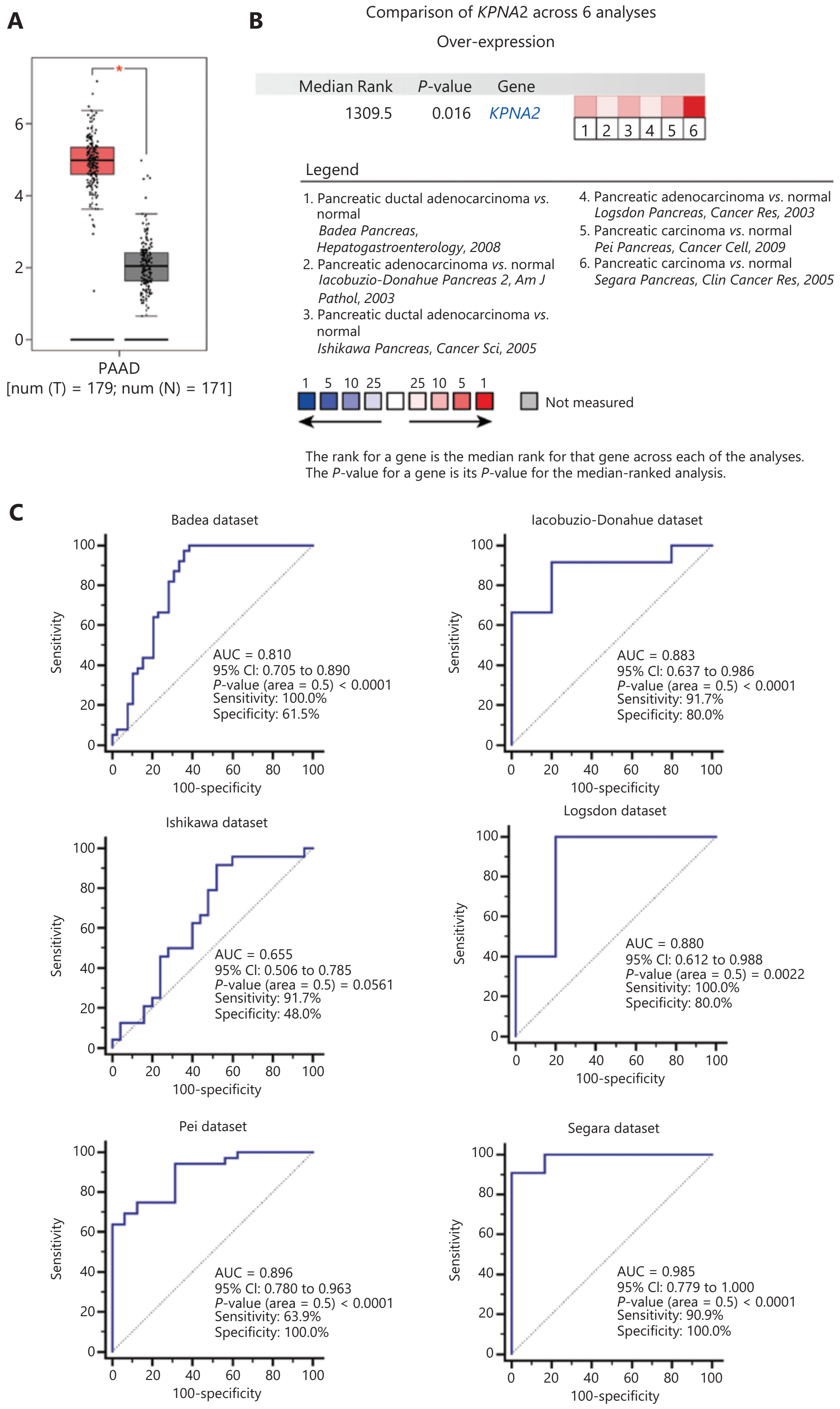

Figure S9 Analyses of *KPNA2* mRNA expression in PA *vs.* normal pancreatic tissues for diagnosis. (A) Analysis of *KPNA2* mRNA expression in PA *vs*. normal pancreatic tissues from TCGA and GTEx datasets. The following settings were used for the analysis: "expression on box plots", "gene = *KPNA2*", "|$\log_2$(FC)| cut-off = 1", "*P*-value cut-off = 0.01", "datasets = PAAD", "log scale = $\log_2$(TPM + 1)", "jitter size = 0.4", and

"matched TCGA normal and GTEx data". (B) Heat map of *KPNA2* expression in PA *vs.* normal tissues with a *P*-value < 0.05 from 6 independent datasets from Oncomine. The 6 datasets include the Badea dataset[4], Iacobuzio-Donahue dataset[3], Ishikawa dataset[9], Logsdon dataset[5], Pei dataset[6], and Segara dataset[2]. (C) ROC curve analysis of *KPNA2* expression with a *P*-value < 0.05 was used to diagnose PA from 5 of 6 independent datasets from Oncomine. ROC analysis of *KPNA2* expression with a *P*-value > 0.05 in the Ishikawa dataset was considered not statistically significant for diagnosis. PA, pancreatic adenocarcinoma; T, tumor; N, normal; **P*-value < 0.01; TCGA, The Cancer Genome Atlas; GTEx, Genotype-Tissue Expression; FC, fold change; PAAD, pancreatic adenocarcinoma; TPM, transcripts per million; ROC, receiver operating characteristic; AUC, area under the ROC curve; CI, confidence interval.

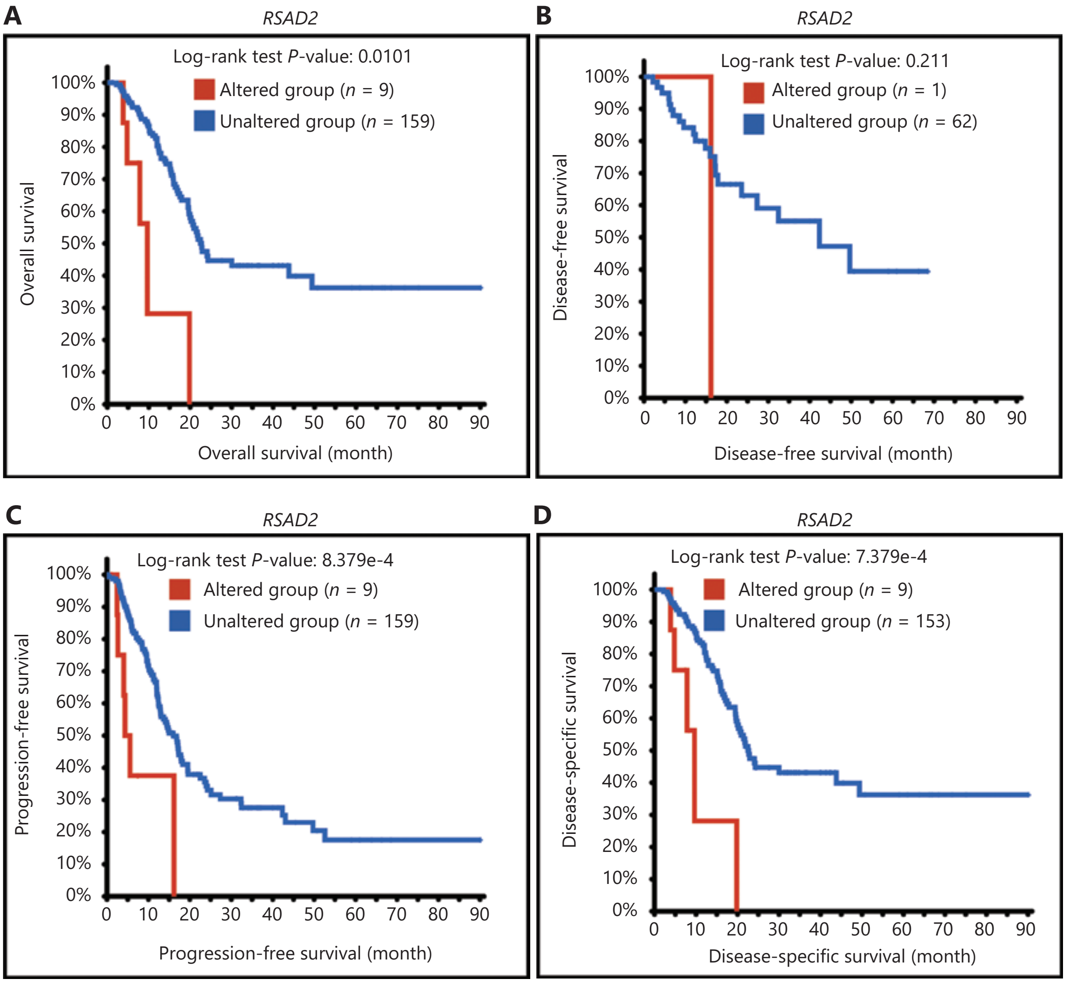

Figure S10 Survival analyses of *RSAD2* alteration performed using cBioPortal. (A) Alterations in *RSAD2* were associated with worse OS (*P* < 0.05). (B) Alterations in *RSAD2* were not associated with worse DFS (*P* > 0.05). (C) Alterations in *RSAD2* were associated with worse PFS (*P* < 0.05). (D) Alterations in *RSAD2* were associated with worse DSS (*P* < 0.05). *n*, number; OS, overall survival; DFS, disease-free survival; PFS, progression-free survival; DSS, disease-specific survival.

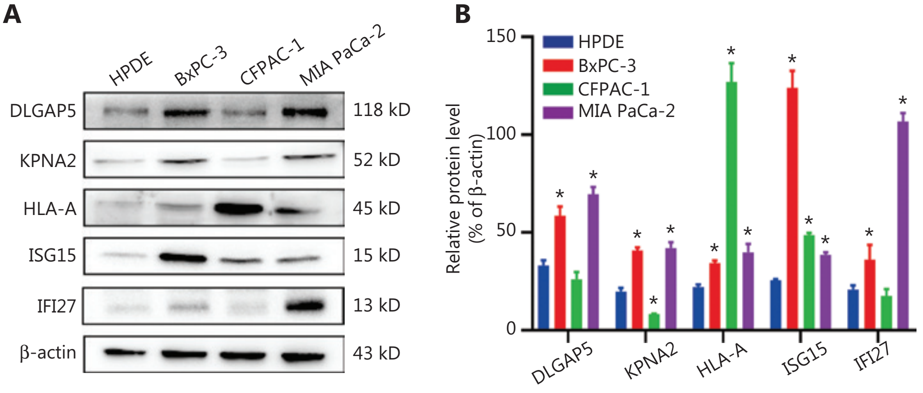

Figure S11 The protein levels of DLGAP5, KPNA2, HLA-A, ISG15, and IFI27 in PA cells. (A) Cell lysates were extracted from a normal pancreatic cell line (HPDE) and 3 PA cell lines (BXPC-3, CFPAC-1, and MIA PaCa-2) and examined by western blot analysis. The results are representative of 3 independently repeated experiments. (B) The relative protein levels (% of β-actin) of DLGAP5, KPNA2, HLA-A, ISG15, and IFI27 in pancreatic cells (cancer *vs*. normal cells). The protein levels of β-actin were detected as the endogenous control. Data are expressed as the means and standard deviations (n = 3). The asterisk indicates that the P-value is less than 0.05. PA, pancreatic adenocarcinoma; n, number.

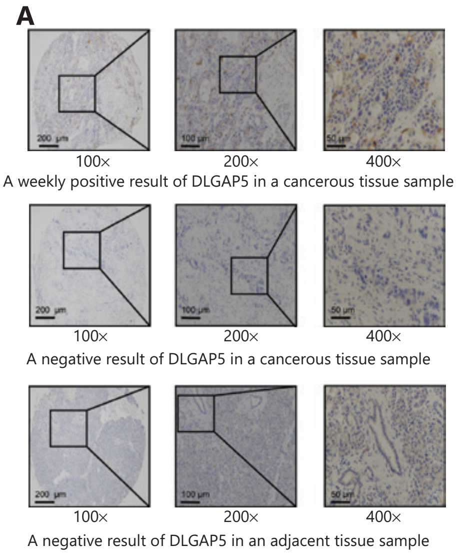

B

Histological type	DLGAP5 IHC score		Total	P-value
	0 [n (%)]	1 to 2 [n (%)]		
Tumor	31 (60.8%)	20 (39.2%)	51	< 0.001
Adjacent	36 (100.0%)	0 (0.0%)	36	

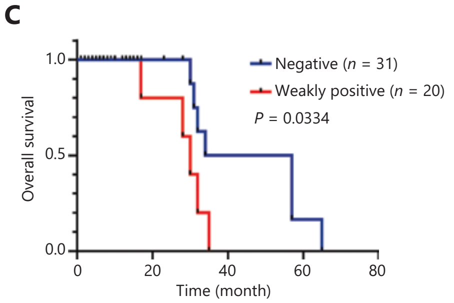

Figure S12 The protein expression of DLGAP5 in PDAC and adjacent noncancerous tissues by IHC. (A) Representative images of IHC staining for DLGAP5 were magnified 100-fold, 200-fold, and 400-fold in the TMA (scale bars: 200 μm, 100 μm, and 50 μm from left to right). (B) Correlation between the protein level of DLGAP5 and histological type. The staining intensity score was determined as no staining (score = 0), light yellow (score = 1), yellow (score = 2), and dark brown (score = 3), while the percentage score of positive cells was determined as < 5% (score = 0), 5%–25% (score = 1), 26%–50% (score = 2), 51%–75% (score = 3), and > 75% (score = 4). An IHC score (staining percentage × intensity) = 1 or 2 was considered a weakly positive result of DLGAP5, while an IHC score = 0 was considered a negative result of DLGAP5. (C) Kaplan-Meier survival analysis of 51 PDAC patients based on negative results or weakly positive results of DLGAP5 expression in tumor tissues. A P-value < 0.05 was considered to be statistically significant. PDAC, pancreatic ductal adenocarcinoma; IHC, immunohistochemistry; TMA, tissue microarray; n, number.

References

1. Zhang H, Pan YZ, Cheung M, Cao M, Yu C, Chen L, et al. LAMB3 mediates apoptotic, proliferative, invasive, and metastatic behaviors in pancreatic cancer by regulating the PI3K/Akt signaling pathway. Cell Death Dis. 2019; 10: 230.
2. Segara D, Biankin AV, Kench JG, Langusch CC, Dawson AC, Skalicky DA, et al. Expression of HOXB2, a retinoic acid signaling target in pancreatic cancer and pancreatic intraepithelial neoplasia. Clin Cancer Res. 2005; 11: 3587-96.
3. Iacobuzio-Donahue CA, Maitra A, Olsen M, Lowe AW, van Heek NT, Rosty C, et al. Exploration of global gene expression patterns in pancreatic adenocarcinoma using cDNA microarrays. Am J Pathol. 2003; 162: 1151-62.
4. Badea L, Herlea V, Dima SO, Dumitrascu T, Popescu I. Combined gene expression analysis of whole-tissue and microdissected pancreatic ductal adenocarcinoma identifies genes specifically overexpressed in tumor epithelia. Hepatogastroenterology. 2008; 55: 2016-27.
5. Logsdon CD, Simeone DM, Binkley C, Arumugam T, Greenson JK, Giordano TJ, et al. Molecular profiling of pancreatic adenocarcinoma and chronic pancreatitis identifies multiple genes differentially regulated in pancreatic cancer. Cancer Res. 2003; 63: 2649-57.
6. Pei H, Li L, Fridley BL, Jenkins GD, Kalari KR, Lingle W, et al. FKBP51 affects cancer cell response to chemotherapy by negatively regulating Akt. Cancer Cell. 2009; 16: 259-66.
7. Buchholz M, Braun M, Heidenblut A, Kestler HA, Kloppel G, Schmiegel W, et al. Transcriptome analysis of microdissected pancreatic intraepithelial neoplastic lesions. Oncogene. 2005; 24: 6626-36.
8. Grützmann R, Pilarsky C, Ammerpohl O, Luttges J, Bohme A, Sipos B, et al. Gene expression profiling of microdissected pancreatic ductal carcinomas using high-density DNA microarrays. Neoplasia. 2004; 6: 611-22.
9. Ishikawa M, Yoshida K, Yamashita Y, Ota J, Takada S, Kisanuki H, et al. Experimental trial for diagnosis of pancreatic ductal carcinoma based on gene expression profiles of pancreatic ductal cells. Cancer Sci. 2005; 96: 387-93.
